# What Would *You* Do? Types of Ethical Challenging Situations Depicted in Vignettes Published in the Veterinary Literature from 1990 to 2020

**DOI:** 10.3390/vetsci9010002

**Published:** 2021-12-22

**Authors:** Anne Quain, Michael P. Ward, Siobhan Mullan

**Affiliations:** 1 Sydney School of Veterinary Science, University of Sydney, Sydney, NSW 2006, Australia; michael.ward@sydney.edu.au; 2 School of Veterinary Medicine, University College Dublin, Belfield, D04 V1W8 Dublin, Ireland; siobhan.mullan@ucd.ie

**Keywords:** veterinary ethics, animal ethics, professional ethics, ethical dilemma, veterinary education, vignette, veterinarian, animal health technician, veterinary nurse, education

## Abstract

Veterinary team members encounter a wide range of ethically challenging situations (ECS) in their work. Inability to resolve ECS in accordance with their values may negatively impact the wellbeing of veterinary team members. We sought to determine the types of ECS described in published ethical vignettes in the veterinary literature. We performed a strategic literature search, followed by a thematic analysis of vignettes published in the veterinary literature from 1990–2020. We identified 567 published vignettes in 544 publications. In the majority of vignettes, the protagonist was a veterinarian (61.6%) and the most common categories of animal involved were dogs (28.0%), livestock in general (10.8%), and cattle (10.6%). The primary type of ECS was coded for each scenario, generating 29 themes. These findings extend knowledge about types of ECS that may be encountered by veterinary team members. These themes can help to inform curricula and better prepare veterinary team members to navigate ECS. They may also highlight factors that contribute to ECS that can be addressed on a broad scale, such as through regulation, continuing professional development, or stakeholder education. Knowing that others may experience similar ECS may help veterinary team members feel part of a moral community.

## 1. Introduction

Ethically challenging situations (ECS) are encountered frequently in veterinary settings [1,2,3,4,5,6,7,8,9]. Inability to resolve ECS in alignment with one’s values may lead to moral stress, moral distress, or moral injury [10,11,12]. Concerningly, moral distress and moral injury may negatively impact wellbeing [11], and may be factors in job turnover and career attrition [13]. Understanding the types of ECS encountered or experienced as particularly stressful by veterinary team members may aid ethical reflection and discussion [14], and may help to ensure that curricula adequately prepare prospective veterinary team members for future challenges.

Reflecting on ECS may reveal systemic factors that can be addressed on a broad scale. This may involve changes in legislation or regulation, development of continuing professional development (CPD) or stakeholder education, cultural change, changes in practice and protocols, or other initiatives. For example, surveys of ECS have identified client financial limitations as an ECS commonly encountered by veterinary team members [1,2,3,9]. This points to a need to improve accessibility of veterinary care [15], to educate animal owners about the costs of veterinary care and the availability of insurance where applicable [16], and to develop sustainable policies for dealing with clients who cannot afford to pay for animal treatment [17]. Developing and implementing these strategies is beyond the capacity of a single, individual veterinary team member. Rather, they require action of employers of veterinary team members, professional associations, non-government organisations, and corporate and government bodies.

Vignettes or case scenarios are commonly used in medical [18] and veterinary ethics teaching [19]. A vignette is defined as “a brief, evocative description, account or episode” [20]. In the context of research, vignettes may be used to assess the impact of contextual factors impacting decision making, for example on the treatment options that veterinarians offer clients with limited finances [17], or whether they are willing to prescribe antimicrobials to sheep or beef farmers without a prior consultation. By incorporating sociocultural and contextual factors, vignettes facilitate application of ethical reasoning in scenarios reflecting ‘real life’ [18]. A number of textbooks employ vignettes to highlight ethical issues in veterinary contexts, facilitate stakeholder identification, provide different perspectives, and prompt the application of different ethical frameworks [21,22,23,24]. Veterinary students in Ireland reported feeling more comfortable discussing someone else’s situation or decision, rather than being required to make an immediate decision about what they might do themselves [19]. After participating in discussions of vignettes, the majority of veterinary students considered themselves better prepared to identify stakeholders and their conflicting interests (79.3%), and find possible solutions to ECS in the future (79.4%). These tutorials also helped students understand the ethical obligations of the veterinary profession (77.8%) and make more informed decisions (80.9%) [19]. Vignettes may also be used to evaluate different ethical approaches [25], assess moral reasoning [26,27] or even inform policy-making [28,29]. Writing brief vignettes on ethics-related themes can also provide a creative outlet, although further research is required to determine whether this helps veterinary team members cope with moral distress [30]. Several veterinary publications, for example, *The Canadian Veterinary Journal* [31] and *In Practice* [32], invite readers to submit vignettes depicting ECS for publication.

We sought to explore published ethical vignettes to gain insight into the types and range of ECS that may be encountered by veterinary team members.

## 2. Materials and Methods

To identify published vignettes, a strategic search was performed in Web of Science (all databases: CAB Abstracts, Current Contents Connect, BIOSIS Previews, and MEDLINE), PubMed, and Google Scholar, carried out between 14 January 2021 and 7 February 2021. Search terms utilised were: (ethic* OR moral) AND (case OR dilemma OR scenario OR vignette) AND (veterinarian OR veterin* OR veterinary technician OR animal health technician OR AHT OR RVT OR veterinary nurse OR RVN). The search was limited to articles published between 1 January 1990 to 31 December 2020, in English. Vignettes that were not available as full texts via Google were sourced via the University of Sydney library or interlibrary loan. Those that were not available as full texts via these sources, or not in English, were excluded.

Web of Science and PubMed entries were exported or manually entered into Endnote for sorting. Duplicates were removed. The remaining Endnote entries were filtered by title and abstract screening, followed by full-text screening to determine whether the article fulfilled inclusion criteria (Table 1). Google Scholar findings were filtered online by the first author using the same inclusion and exclusion criteria.

Rigorous qualitative research is acknowledged to be “context-bound, positioned and situated” [33]. In other words, analysis of qualitative data involves interpretation. Rather than being viewed as a threat to knowledge production, researcher subjectivity is viewed as a resource, with researchers taking an active role in data production [33]. Research questions, study design, and methods of analysis are inextricably linked to the perspectives through which the researchers view the world [34]. TA is ultimately “an *interpretive* activity undertaken by a researcher who is situated in various ways, and who reads data through the lenses of their particular social, cultural, historical, disciplinary, political and ideological positionings” (original emphasis) [35]. To this end, it is considered best practice to outline their own position and background, even briefly [35,36].

The first author is a companion animal veterinarian, practicing as a primary accession veterinarian within metropolitan, urban, and regional areas within Australia, and a lecturer in the Sydney School of Veterinary Science. In teaching veterinary ethics, she draws upon both published surveys documenting the ECS encountered by veterinary team members, as well as published vignettes, some of which she contributed. The latter appear in this analysis. Her interest in the types and stressfulness of ECS stems from personal experience and discussions with colleagues and DVM students.

The second author is a veterinarian, lecturer in epidemiology and public health, and a researcher at the Sydney School of Veterinary Science. His veterinary practice experience is derived exclusively from government practices as a field veterinarian. He teaches research methodology to first-year DVM students and coordinates third-year DVM student research projects. The latter includes screening and checking research projects for ethics (animal and human) approval and best research practice and advising students on approaches to researching veterinary topics.

The third author is a veterinarian, researcher, and lecturer in veterinary ethics at University College Dublin. She has a long-standing interest in veterinary ethics, starting as a student and continuing through practice and into teaching. She instigated and coordinated a vignette-based series, ‘Everyday Ethics,’ in the UK veterinary journal *In Practice* for 10 years and 100 issues. Some of these vignettes were submitted by readers, others were proposed by potential responders, and some were written by the second author. All of these vignettes appear in this analysis.

The Endnote library was exported into NVivo12 Plus (QSR International). Data were analysed using principles of thematic analysis (TA) using an inductive approach aligned with codebook TA [37].

The analytical process involved six stages. Firstly, the first author read each vignette at least three times to familiarise herself with the vignettes. Secondly, initial codes were generated. Each vignette was coded inductively for semantic themes, employing a realist approach without a pre-existing theoretical framework. An iterative approach was used. Each vignette was initially coded three times according to the role of the protagonist, the type of animal involved, and the primary ECS described. Where vignettes involved multiple protagonists or species, the vignette was coded according to the first mentioned. For example, if the vignette stated, “you are a veterinary technician…” or “Dr X is a veterinarian”, it was categorised according to the role “veterinary technician” or “veterinarian” respectively. If a vignette did not specify a role but referred to a protagonist who had made a diagnosis or performed surgery, the role was classified as “veterinarian”. If the vignette did not specify a role or posed an ethical challenge for which the role within a veterinary team was not relevant—for example, where a role was not specified and the vignette raised a general question whether a type of animal use is acceptable—the role was coded “not applicable”. Where there was no protagonist, the vignette was coded “not applicable” for the role. Where the vignette did not refer to any animal (for example, those concerned with collegial relations), it was coded “not applicable” for the animal category. Types of ECS were initially coded according to ECS identified in surveys, as shown in Table 2. Where an ECS could not be coded according to an existing code, a new code was generated.

Thirdly, initial themes were generated. To facilitate initial coding of semantic themes associated with the type of ECS, we identified surveys and reviews focused on determining the type, frequency, and/or stressfulness of ECS encountered by veterinary team members. From each of these, we compiled a list of specific types of ECS, either directly from the survey where this was available, or in other cases, key ethical challenges identified. A codebook approach is often utilised with large datasets, providing structure that offers some efficiency in analysis [38]. In addition, new themes were generated through inductive data engagement and analysis [35], the latter overlapping with reflexive TA [33].

The list of codes was examined to identify clusters of codes and complex codes which were grouped together as themes deemed to best represent the data. Themes were reviewed for both internal coherence and distinctiveness from other themes. This involved regularly re-reading all coded extracts from each theme. Where extracts did not fit a theme, these were either reallocated to a more appropriate theme or allocated to a new theme. The first and third authors discussed coding and initial generation of themes.

The fourth and fifth stages—refining themes and developing a thematic map, and defining and naming themes—were performed concurrently, and involved further discussion between all authors. The sixth and final stage involved construction of a table describing key ECS within each theme. We counted the number of vignettes coded for each theme, to indicate the prominence of themes relative to one another. While this is not typical of a TA approach [36], we chose this approach due to the large breadth but relatively shallow depth of data collected, as has been done in other studies, including veterinary surveys involving large numbers of free-text responses [39].

## 3. Results

### 3.1. Development of Initial Codebook

We identified nine publications, comprising seven surveys, one committee report, and one policy Delphi listing key ethically challenging situations encountered by veterinarians. Of these, seven listed specific or key ethically challenging situations, either in a questionnaire or as a summary (see Table 2). The policy Delphi provided summaries of ECS in rounds two and three [29]. As the ECS outlined for the second round was more closely aligned with ECS depicted in the surveys, we utilised the summary from the second round in coding.

Two surveys “deliberately refrained from giving concrete examples or a given definition of ‘morally challenging situations’” [6,8]. These surveys of German farm veterinarians (*n* = 123) and Bavarian veterinary officers (*n* = 81) asked respondents to report the frequency of ECS in broader terms, notably: “1. I wasn’t sure what was the morally right thing in this situation; 2. I was sure what was the morally right thing to do, but I could not, or only partially, implement it; 3. My personal moral convictions contradicted the legal requirements; 4. No matter how I decided…there were always weighty moral reasons against this decision; 5. I knew what would have been morally right, but the implementation would have meant a considerable extra effort for me.” Because of the broad nature of these ECS in comparison to those listed the other publications, we did not utilise these in coding.

### 3.2. Vignettes

**Figure 1:**
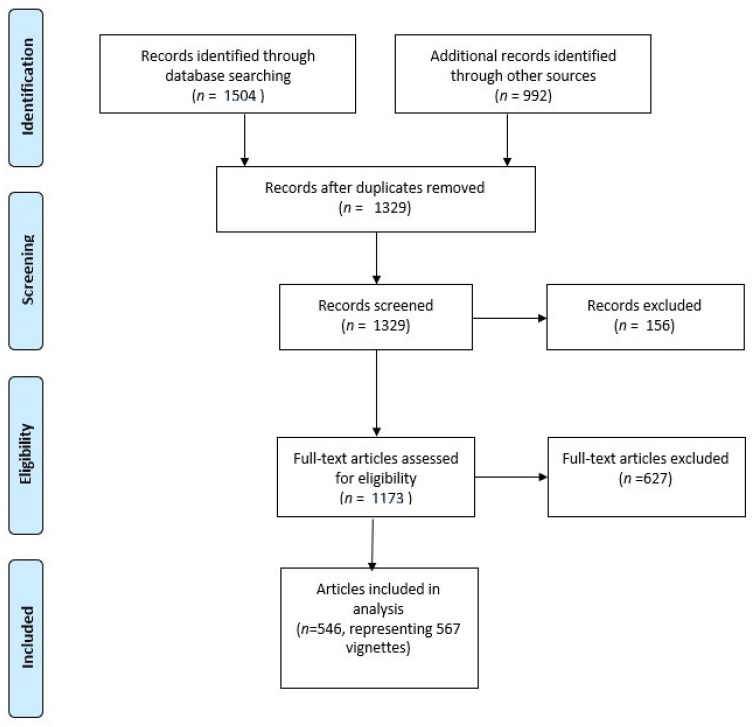
Flow diagram of literature searches [41].

Web of Science (all databases) and PubMed searches returned 862 and 641 records, respectively, a total of 1503 records (Figure 1). A Google Scholar search yielded 992 hits. At this stage there were a total of 2495 records, of which 1166 were duplicates. Therefore, 1329 records were screened. After screening, based on the title, abstract, or full text, there were 546 articles containing 567 vignettes (for bibliographic information, see Supplementary Material S1). Figure 1 provides a flow diagram of the literature searches.

The majority of vignettes came from two sources: the *Canadian Veterinary Journal* (61.0%, *n* = 346) and *In Practice* (26.1%, *n* = 148), both publications aimed at veterinarians. The *Australian Veterinary Journal* accounted for another five vignettes (0.9%). Vignettes featured in journal articles that were designed for veterinary students and veterinarians accounted for 4.2% [19,29]. A smaller number of vignettes appeared in publications targeted specifically at veterinary nurses and animal health technicians, including a vignette-based textbook (*Exploring the Grey Zone*) [23] (4.8%, *n* = 27); *Veterinary Technician* (1.9%, *n* = 11), *The Veterinary Nurse* (0.5%, *n* = 3); and *Veterinary Nursing Journal* (0.5%, *n* = 3). Vignettes were contributed by a combination of panels (for example, the *Canadian Veterinary Journal* noted that cases would be provided by a panel comprising large and small animal clinicians [42]), column, or journal editors (some of whom polled readers in online discussion forums [43,44]), and readers [42,44], some of whom chose to remain anonymous. The exceptions were articles which described the development of vignettes on the basis of focus groups, literature reviews and other sources [19,29], and a vignette-based textbook for which cases were “purposely created…to represent the more realistic scenarios in which there is often more than one correct course of action…” [23].

**Figure 2:**
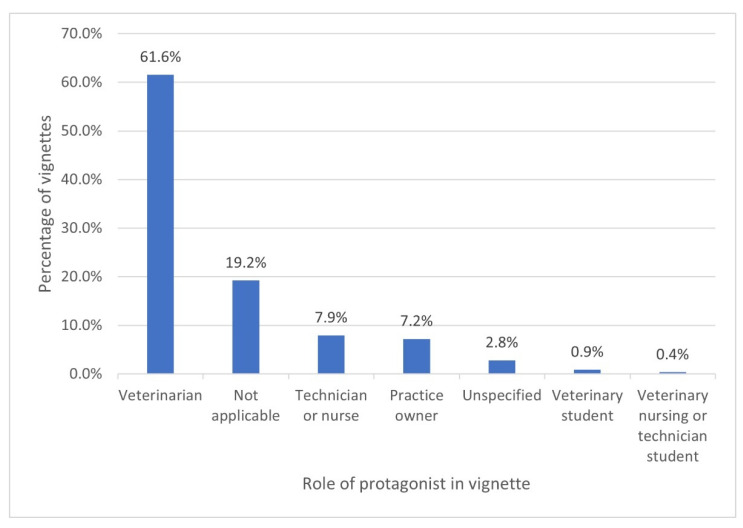
Bar chart depicting the role of the protagonist in ethical vignettes (*n* = 567).

The role of the protagonist in each vignette is presented in Figure 2. The majority of vignettes described ECS faced by veterinarians (61.6%, *n* = 349). In addition, where the protagonist was a practice owner (7.2%, *n* = 41), veterinarian practice owners were specified in the majority (87.8%, *n* = 36) of these vignettes. The next most frequent category was “not applicable” (19.2%, *n* = 109).

**Figure 3:**
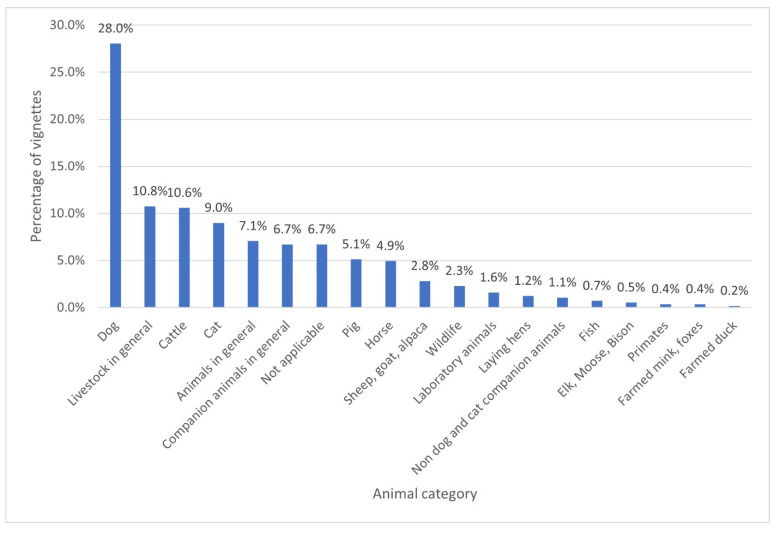
Bar chart depicting the primary category of animal in ethical vignettes (*n* = 567).

The categories of animals featured are presented in Figure 3. The most frequent category was dogs (28.0%, *n* = 159), followed by livestock in general (10.8%, *n* = 61), cattle (10.6%, *n* = 60), cats (9.0%, *n* = 51), animals in general (7.1%, *n* = 40), and companion animals in general (6.7%, *n* = 38). Some cases did not feature an animal (6.7%, *n* = 38), for example those focused exclusively on collegial relations.

Themes generated from the types of ECS described in the vignettes are described in Table 3. In total, 29 themes were generated.

## 4. Discussion

Analysis of published veterinary ethical vignettes reveals that veterinary team members may encounter a broad range of ECS in their work. The fact that we identified 567 vignettes comprising 29 themes confirms that many ECS are not unique, which may give veterinary team members a sense of moral community [45].

The sources of vignettes varied, and included panels, column or journal editors, journal readers, researchers, and book authors. It was not possible for us to determine the degree to which vignettes reflected the actual experiences of veterinary team members, if at all. Indeed, some vignettes were developed deliberately to provide an example of reportedly common scenarios [29] or to provoke ethical reflection [23]. Nonetheless, as veterinary team members ourselves, we found the vignettes plausible and realistic. The protagonist of the majority of vignettes, also accounting for the majority of respondents to surveys on ECS, was the veterinarian in clinical practice. This reflects the reality that the majority of veterinarians in western countries work in clinical practice [46,47,48,49,50,51]. Historically, veterinarians worked sole charge, however, veterinarians now tend to work within teams incorporating paraprofessionals [52]. Additionally, veterinary nurses and animal health technicians have undergone professionalisation, including the introduction of professional associations, a register, a code of ethics, disciplinary proceedings, and CPD [52,53]. Veterinarians are not alone in experiencing ECS in their work, nor are they the sole decision makers in their workplace. To reflect the reality of veterinary workplaces, it may therefore be helpful to develop more vignettes that feature non-veterinarians, or veterinary teams, as protagonists.

A perspective missing almost entirely is that of the client, animal owner, or guardian. While vignettes serve a purpose in the education of veterinary team members about ECS they may encounter, the client—where featured—is almost invariably portrayed as the source of the ECS, or a barrier to its resolution, rather than as someone who may be experiencing ethical challenges themselves. In portraying clients in this way, there is a risk of failing to consider their perspectives and interests. One vignette describes a veterinarian’s assessment of a farm dog that they are called out to examine, and diagnoses a fractured left femur [54]. In the scenario, the veterinarian offers the options of surgical repair or euthanasia, leaving the dog with analgesics while the owners decide. The owners choose neither, instead nursing the dog at home, and in time the dog makes a complete clinical recovery. According to the vignette, the protagonist is “…shocked that the dog was left with a broken leg, shocked that it is now running around at [their] feet” and realises that they “should have followed up to ensure that the dog was euthanased” [54]. However, the dog’s recovery and subsequent “good life” move the veterinarian to ask, “Was offering surgery or euthanasia the only appropriate options to suggest in this case?”.

After reading the ethicist’s response to the vignette, the owners of the dog depicted in the vignette wrote to the journal, ostensibly in defence of their veterinarian. However, their letter provides insight into the factors that impacted their decision making—not discussed with their veterinarian at the time—including their own assessment of the dog’s pain and beliefs about analgesia and animal welfare, and financial constraints they faced “as parents of five children and living solely on a farm income” [55]. Further discussion between the veterinarian and the clients may have revealed further constraints and opportunities and led to the provision of alternative options—such as splinting, cage rest, and extended analgesia—along a spectrum of care [56,57]. This correspondence also demonstrates that, despite providing contextual information, vignettes do not provide all relevant information. It is a reminder that, in addressing ECS, veterinary team members should consider the information that may be missing, or sources of additional data that may help characterise the ECS and develop an appropriate response.

Companion animals (“dogs”, “cats”, “companion animals in general”) and livestock (“livestock in general” and “cattle”) accounted for the majority of species or category of animal depicted in vignettes, probably because veterinary team members are most likely to encounter these groups of animals. The prevalence of companion animal-related vignettes may reflect the reality that, in most western countries, the majority of veterinary teams care exclusively or mostly for companion animals or small animals [46,47,48,49,50,51]. Dogs may have featured more prominently in vignettes due to a perception that they form strong affiliative bonds with humans, who are responsive to the canine gaze [58]. Dog owners may also have stronger bonds than cat owners, be more likely to seek veterinary attention for them, and consider more costly (and potentially more involved) intervention when compared with cat owners [59,60]. Dogs may feature more prominently than cats as owners of cats may avoid taking them to veterinary clinics due to “feline resistance” to carriers or transportation, and fearful behaviour in veterinary settings [61].

Aside from the companion animal bond, companion animals may have featured more prominently in vignettes due to a broader spectrum of treatment options (introducing more variables to consider in decisions around euthanasia) [62], and ethical challenges associated with advanced veterinary care [63]. This focus of the veterinary profession on companion animals has been criticised as socially irrelevant in the face of the growing human population, stress on global resources, and increasing threats to biosecurity [64].

Livestock featured heavily in themes such as “what forms of animal use are acceptable”, “ensuring food safety, food security and biosecurity”, “how to balance animal productivity with animal welfare”, and “slaughter and killing of farm animals”. Vignettes that featured cattle primarily (10.6%, *n* = 60) were more likely to feature dairy cattle (55.0%, *n* = 33) than beef cattle (23.3%, *n* = 14) or unspecified (21.7%, *n* = 13). This may reflect increasing public concerns about practices such as culling of male calves and the separation of calves and cows [65].

Horses were specifically featured in less than 5% of the vignettes. This is somewhat surprising given increasing concerns about the welfare of working equids, the use of horses in sport and recreation (particularly in relation to breeding, potential conflicts of interest of veterinarians attending to sporting horses, the use of whips and nosebands, and fate of surplus animals) [66,67,68,69,70,71,72]. It is possible that such issues are believed to be beyond the remit of veterinary team members, who have a largely clinical focus, as they raise broader issues around animal use.

The relative prevalence of these species may reflect the change in the focus of clinical veterinary practice in the 20th and 21st century. This focus shifted from the horse at the beginning of the 20th century, to the dairy cow, to companion animals from the middle of the 20th century to the present day [73].

Animal categories including “sheep, goats and alpacas”, “wildlife”, “laboratory animals”, “laying hens” and “non dog and cat companion animals” featured in less than 5% of vignettes, while “fish”, “elk, moose, bison”, “primates”, “farmed mink and fox” and “farmed duck” featured in less than 1% of vignettes. This may reflect the relatively small number of veterinary team members working with these categories of animals, rather than reflecting the range of ECS they encounter. This aligns with a review of papers presented at the World Association for the History of Veterinary Medicine, which found that fish, wildlife and exotic species were among the least commonly discussed [73].

Given concerns about the impact of occupational stressors on the wellbeing of veterinary team members [74,75,76,77,78,79,80,81], we believe that it is important to equip current and prospective veterinary team members with knowledge and skills to successfully navigate ECS. We believe the themes generated from these vignettes, using published surveys of ECS, provide a useful foundation. For example, in knowing that veterinary team members may encounter ECS relating to the client who refuses a recommendation (for example, a recommendation to put at overweight dog with a mammary mass on a diet [82] or to perform a caesarean on a heifer [83]), or does not care appropriately for sick animals per your instructions [84], educators, professional associations, organisations, and employers may find it beneficial to provide opportunities for training in communication and conflict management [85]. For example, learning motivational interviewing techniques may improve communication with farmers around herd health management [86].

Veterinary team members support and often engage in animal use themselves (for example, keeping of companion animals, utilising animals in education and research, farming, or consuming animals). They also engage with colleagues and clients with diverse and dynamic views about what forms of animal use are acceptable. Numerous vignettes raised the question of what forms of animal use are acceptable, suggesting the need for veterinary team members to reflect on their own views and consider relevant evidence, for example, from animal welfare science. This is reflected in the OIE recommendations on Day 1 veterinary competences, which specify that veterinarians should “provide leadership to society on ethical considerations involved in the use and care of animals by humans” (2.9, Veterinary Legislation and Ethics) [87].

It is important to reflect on factors that may have influenced the development of themes presented here. For example, conflict between the interests of animals and the interests of their owners. Rollin stated that the “fundamental question of veterinary ethics” is “to whom does the veterinarian owe primary obligation—animal or owner?” [21]. Tannenbaum described the veterinarian as the “servant of two masters”—human clients, on the one hand, and animal patients on the other:

“…veterinarians are expected to serve *both* their human clients and animal patients. Indeed, they are often called upon to serve as an advocate of both parties’ interests, even when these interests conflict. Thus, veterinarians will often speak out on behalf of the animal, telling the client how the animal feels or is likely to fare, and indicating what is or is not in its interests. At the same time, veterinarians are often asked to be advocates for their clients’ interests—to know, for example, what would make the pet owner happy, the racehorse owner wealthy, or the researcher successful.”

[88] (p. 146)

However, the conception of ECS as occurring within this triadic relationship between veterinarian, client/owner, and patient overlooks the reality that veterinary team members rarely work in isolation, are often employed, and may not have ethical responsibility withcomplete decision-making autonomy. This predicament has been raised in the context of other professions, for example engineering, where engineers are expected to do what is right and prevent what they recognise as wrong. This may lead to conflict with colleagues and employers:

“The engineer is usually working with a team, and he or she first has to persuade the collaborators to modify or even stop a project because of ethical concerns. Moreover, the engineer is dependent on the employment contract he or she has signed towards the employer. Through this contract the engineer becomes subject to directives, and thus renounces his or her personal autonomy as far as professional work is concerned, and he or she undertakes to keep secret any internal business information. So, on principle the moral responsibility of the individual engineer is cut by industrial law. Even if, meanwhile, in some countries refusal to work and whistle-blowing are legally accepted in cases of serious concern, the engineer involved is usually risking his or her career. Engineering ethics, in terms of individual responsibility, in the borderline case is forcing the engineer to play the moral hero, a role that is neither desirable nor realistic”.

[89]

Yet the pervasiveness of the conception of the veterinarian as a “moral hero” in veterinary settings may explain why the majority of vignettes feature a veterinarian as the protagonist, why major surveys regarding ECS in veterinary settings have focused on veterinarians [1,2,3,4,6,7,8], rather than non-veterinary team members, and why many of the ECS about which veterinarians have been surveyed involve conflict between the interests of the client and those of the animal patient [1,2,3,4,6,7,8].

As an alternative to the veterinarian–client–animal triad, Durnburger talks about “a triangle within a square”: the triangle consisting of the veterinarian, animal, and client, situated within a square including politics and legal requirements, society and its expectations, other veterinarians in different roles (including colleagues, supervisors, employees, and competitors), and veterinary officers (as the essential supervisory body) [7]. It may be that the use of such a model may alter the way veterinary team members perceive and experience ECS.

Similarly, veterinary team members are guided in their daily work by codes of professional conduct and animal welfare legislation, but interpretation is not always easy, and laws and regulations are not uniformly enforced [90]. Additionally, legislation may constrain professional judgement, preventing veterinary team members from acting in alignment with their values. For example, Portuguese legislation preventing euthanasia of unowned companion animals except in cases of intractable pain and suffering, was perceived as a potential barrier to ethical behaviour by veterinarians [28]. Vignettes coded under the theme “animal welfare governance” suggest a need for resources to help veterinary team members understand how animal welfare and veterinary legislation is developed, what their obligations are, anticipating and managing unintended consequences, understanding limitation, and how legislation is updated or changed. Educators and professional bodies may need to ensure they provide up-to-date, relevant training that goes beyond an overview of animal welfare governance and describes implementation. Workplaces and professional bodies may be able to provide clear pathways for seeking appropriate advice.

Veterinarians and other veterinary team members, including registered veterinary nurses and animal health technicians, are required to make professional judgements and be able to justify these according to sound principles. According to the Royal College of Veterinary Surgeons “Day 1 Competencies”, veterinarians, for example, “must be able to think through the dilemmas they face when presented with conflicting priorities and be prepared to justify the decisions they make. As well as decisions relating to individual patients, animal groups, populations of animals and clients, veterinary surgeons must take account of the possible impact of their actions beyond the immediate workplace, for example, on public health, the environment and society more generally” [91].

### Limitations

For pragmatic reasons, each vignette was only coded according to what the authors perceived as the primary ECS depicted, yet vignettes varied in complexity (as real-world ECS may vary in complexity), with some depicting multiple, often overlapping ECS which could have been coded differently. For example, a vignette describing a “recently qualified veterinary nurse” who has “noticed that some of the procedures used in the practice do no concur with what she was taught”, specifically, procedures that the nurse feels are below the standard of what she was taught [92], was coded as “standard of care”. However, the vignette also notes that the nurse raised concerns with veterinarians in the practice, only to be “brushed off with flippant remarks” [92]. Therefore, the vignette could have been coded as “collegial relations and wellbeing of veterinary team members”. In this instance, we deemed that “standard of care” was the primary ECS raised. That said, vignettes were often presented as if they contained a single ECS. For example, many vignettes in the *Canadian Veterinary Journal* typically close with a question, in bold, posed as a single ethical dilemma. For example, “If the behaviour of caged rodents can never be representative of human behaviour, is such experimentation ever justified?” (original bold) [93].

It is possible that published vignettes may not reflect ECS most commonly encountered by veterinary team members. This may be because veterinary team members have become desensitised to common ECS, or that they have established workable approaches to deal with common ECS [1]. In medical training there is a tendency to focus on case studies involving less common but perhaps more extreme ECS: “When residents select cases they tend to unduly emphasise life support and decisions regarding resuscitation and ignore the much more common cases, such as mild hypertension; teaching residents to recognise the ethical components of such everyday cases is an important goal of our program as well” [94]. While we assumed that, collectively, this body of vignettes is reasonably representative of ECS encountered by veterinary team members, it is therefore possible that at least some vignettes represent outliers. It is important for readers to note that the numbers of vignettes coded under each theme cannot indicate the frequency of the particular types of ECS represented in that theme encountered in veterinary settings. For example, in this study, the fourth most frequently coded them was “What should veterinary team members do when clients breach welfare laws or regulations?” However, in a survey of 540 veterinarians, “suspected patient/animal abuse” was the least frequent ECS encountered, but the most morally significant [2]. Those composing and selecting vignettes for publication may be motivated to write about a topic that is more likely to interest a reader, rather than more commonly encountered ECS.

Our search strategy omitted vignettes from the grey literature, a potentially rich source of ECS encountered by veterinary team members. Inclusion of grey literature, including newsletters, research and committee reports, conference proceedings and abstracts, dissertations and even online forums, may reduce publication bias [95]. It is particularly helpful in the context of a paucity of information in peer-reviewed literature [95]. However, there are several disadvantages to using the grey literature, including the challenge of developing a sensitive and specific search strategy, and lack of consistency in title and indexing information [95]. We elected not to incorporate a grey literature search for these reasons.

The majority of vignettes were published prior to the COVID-19 pandemic, during which veterinary team members encountered novel ECS including decisions about what counts as an essential veterinary service, conflict between the wellbeing of household members and professional role, and whether to perform non-contact vet visits [9]. For some veterinary team members, widespread shortages of personal protective equipment (PPE), hand sanitiser, ventilators, and other equipment in human healthcare settings rendered their use in veterinary settings ethically challenging [96]. We only found one vignette that explicitly referred to the pandemic [97]. There may be a substantial lag time between encountering a new or novel ECS and writing about it, in which case we may see more vignettes dealing with pandemic-associated ECS in the future.

Thematic analysis is not performed in an epistemological vacuum. A realist approach to thematic analysis assumes a predominantly unidirectional relationship between meaning, experience, and language. However, this may overlook the diverse sociocultural contexts and structural conditions that underpin these scenarios in the first place [98]. Due to publication and sampling bias, it is most likely that veterinary team members from relatively well-off, English-speaking contexts would be more likely to contribute vignettes to the publications that invited these. We coded vignettes according to the primary ECS that we identified; however, this may not reflect the ECS as experienced by the author. Published vignettes may have undergone editing following submission to the extent that they may no longer accurately reflect the emphasis originally intended. In the medical literature, case analysis is acknowledged to be “prone to misunderstandings and misinterpretations” [18], and is highly dependent on the quality and extent of information provided.

What counts as an ECS may vary between veterinary team members. The vignettes analysed in this study were presented as ethical challenges or ethical dilemmas, based on an underlying assumption that they would be experienced as such. However, one study found variation among veterinarians as to whether a particular scenario was experienced as ethically challenging (a ‘dilemma’) or not at all [3]. Whether something is experienced as an ECS may depend on interaction between characteristics and perspectives of those involved and contextual factors. It may be of interest, in future studies, to survey veterinary team members about which vignettes—or aspects of vignettes—they find ethically challenging, and why that is the case.

While vignettes provide contextual factors that may complicate ECS, it is impossible to depict every iteration of an ECS that a veterinary team member may encounter. Durnburger found that a key dilemma faced by German farm-animal veterinarians was conflict between personal convictions and external constraints [8]. We agree that it is important to equip veterinary team members to recognise and address these broader conflicts. Vignettes may facilitate application of ethical reasoning and problem solving.

The authors acknowledge that reliance on case-based teaching in ethics may overemphasise the weight of isolated decisions of individuals, while underplaying the broader institutional and social contexts that create and shape ethical challenges [94]. It is important that those utilising vignettes in the teaching of veterinary ethics are attentive to the possibility that the appropriate response may require systemic change that transforms the options available, or allows an ECS to be avoided [94].

## Figures and Tables

**Table 1:** Inclusion and exclusion criteria for screening search outputs.

Criteria	Inclusion	Exclusion
Outcome	Vignette (a brief, evocative description or scenario)	Not a vignette
Population	A vignette written with a veterinary team member (veterinarian, animal health technician, veterinary nurse, or associated ancillary staff, including those working in laboratory, academic, and regulatory settings) as a protagonist, and/or published in a journal or publication written for veterinary team members, depicting an ethical challenge or ethical dilemma.	Vignette is not written with a veterinary team member (veterinarian, animal health technician, veterinary nurse, or associated ancillary staff, including those working in laboratory, academic, and regulatory settings) as a protagonist, and/or published in a journal or publication written for veterinary team members, does not depict an ethical challenge or ethical dilemma, or is developed for a stated purpose other than to depict an ethical challenge or ethical dilemma.
Publication type	Vignette Article containing a vignette or vignettes depicting an ethical challenge or ethical dilemma.	Commentary on a vignette Systematic review Clinical case report/case series Randomised controlled trials Cohort studies
Availability	Available through the University of Sydney Library or interlibrary loan.	Unable to obtain full text of vignette.
Language	English	Language other than English.

**Table 2:** Specific or key ethically challenging situations (ECS) encountered in veterinary settings explored in published surveys/reviews, utilised for initial coding of vignettes.

Study	Participants	Practice Type	Source of ECS	Specific or Key Ethically Challenging Situations Listed in Publication
Batchelor and McKeegan [1]	*n* = 58	Small animal Large animal Equine	“Common” scenarios based on review of literature.	Convenience euthanasia of a healthy animal Financial limitations of the client restricting the treatment options The client wishing to continue treatment despite compromised animal welfare/quality of life
Crane et al. [2]	*n* = 540	Small animal Large animal Mixed Specialist	Focus group of 11 veterinarians (3 rural based, 8 urban based); review of literature.	Working in a situation where the owner would not pay for the recommended treatment Carrying out the owner’s wishes that were not in the best interest of the animal patient Balancing the welfare of the human client with the welfare of the animal patient Assisting other veterinarians who they believed were providing incompetent care Performing euthanasia in general Performing euthanasia for reasons they did not agree with Suspected patient/pet abuse.
Magalhaes-Sant’Ana [29]	*n* = 20	Veterinary practitioners, veterinary inspectors, veterinary nurses in Ireland.	Three-round policy Delphi with vignette methodology.	Adequate food safety standards (e.g., to prevent manipulation of meat inspection reports) Responsible disease eradication programs (e.g., to prevent inappropriately influencing the interpretation of a tuberculosis test result) Responsible casualty slaughter certification (e.g., to prevent incorrectly certifying an animal as being fit for transport) Responsible veterinary exports certification (e.g., to prevent certifying a herd with an unknown disease status) Responsible animal insurance schemes (e.g., to prevent client pressure to change vaccination date) Responsible use of social media by veterinary professionals (e.g., to prevent posting a picture of an animal without client’s consent) Working relationships between veterinarians and veterinary nurses (e.g., nurse being asked to do something that conflicts with his/her ethical values) Guidance on referrals and second opinions (e.g., to prevent failing to refer an animal to another colleague) Guidance on continuing veterinary education (e.g., to prevent asking for the certificate from a seminar you paid for but did not attend) Responsible clinical research and teaching involving animals (e.g., vet students taking samples from owned animals for their Master of Veterinary Medicine) Performing convenience animal euthanasia (e.g., putting down surplus foals) The provision of 24 h and emergency veterinary care (e.g., to prevent lack of adequate overnight care) Prudent prescription and administration of veterinary medicines (e.g., to prevent excessive use of antibiotics) The role of veterinary professionals in unregulated animal fairs, races and shows (e.g., to prevent failing to report abuse to animals) Responsible advanced treatments in small animal medicine (e.g., pet cloning or cat kidney transplants).
Kipperman et al. [3]	*n* = 484	Small animal (including: shelter medicine, mobile, emergency, feline only) Mixed Specialist Academic Non-listed	Not specified.	Client financial limitations compromising the quality of the care the respondent could provide for the patient Euthanasia requested because of economic limitations, which the respondent believed was due to lack of financial means Euthanasia requested where the respondent believed the client had the financial resources, but was unwilling to pay for treatment Euthanasia requested because of client convenience Euthanasia requested without a reason, but the respondent felt it was not in the animal’s best interest Treatment requested when a patient’s prognosis was hopeless or recovery is very unlikely Client unwilling to treat or euthanase a patient that the respondent believed was terminal and suffering Having to perform empirical therapeutic trial instead of diagnostic testing because of costs or owner preference
Moses et al. [4]	*n* = 889	Small animal Equine Food animal Exotic animal	Not specified.	A conflict of opinion with pet owners about how they wished to proceed in the treatment of their pets/ Pet owner’s attitudes or beliefs about treatment made it difficult to provide the care the respondent thought was appropriate Being asked to do something in their clinical practice that felt to the respondent like the wrong thing to do A case where the respondent felt like they could not do the “right thing” Receiving an inappropriate request for euthanasia Managing cases where the respondent felt that a pet owner requested treatment when the respondent considered those efforts to be futile/Refuse to provide treatment that the respondent felt was futile Recommending euthanasia to pet owners if they did not bring up the topic Recommending euthanasia to pet owners when they already said they would not consider it Being asked to do things that are outside of the respondent’s skill set for financial or other reasons Disagreements with other veterinarians about how best to manage a case the respondent shared with them Disagreements with non-veterinary staff members about how best to proceed with a clinical case Feeling conflicted about prioritising the needs of animal owners over patients
World Small Animal Veterinary Association [40]	*n* = 8	Small animal	Compiled by the animal welfare guidelines group.	The decision to assist in treatment and breeding of animals with extreme traits associated with health problems Whether euthanasia is acceptable and, if it is, when and how should it be performed Whether the veterinarian should perform cosmetic or convenience surgeries such as ear cropping, tail docking, declawing, or debarking Whether to treat an animal to extend their quantity of life, and how this impacts quality of life Whether to use animals for blood transfusions or as sources of organs for transplants, which animals to source these from and how to treat source animals When to breach client confidentiality in the interests of animal welfare, human welfare, or public safety How to manage cases where abuse, mistreatment or neglect of an animal is suspected The decision to surgically spay or neuter an animal Management of inappropriate or inadequate feeding of animals.
Lehnus et al. [5]	*n* = 183	Veterinary anaesthetists (including Diplomates, residents, and nurses or technicians performing anaesthesia)	Not specified.	Ethical disagreement with colleagues regarding whether decisions are in the best interests of the patient Performing anaesthesia against one’s conscience Financial constraints which limit the type of treatment that can be given (where owner wishes to continue treatment within their means) Ethical concerns around modern intensive care medicine

**Table 3:** Themes generated from a review of ethical vignettes published in the veterinary literature from 1990 to 2020, with a summary of key ethically challenging situations described within each theme.

Theme	Key Ethically Challenging Situations (ECS) Described within Theme	Number of Vignettes Coded
How to manage a client who refuses a recommendation or does not adhere to advice	How do veterinary team members manage clients who refuse to euthanase an animal with poor welfare or deteriorating quality of life? What if a client refuses to follow advice in situations where public health is at risk? How should a veterinarian respond if a client refuses to allow them to examine animals on a property that require veterinary attention? What if a client wishes to pursue inappropriate, high-risk, or potentially harmful treatment? How do you manage a client who does not adhere to instructions?	43
What forms of animal use are acceptable?	Are some forms of animal use unacceptable? On what basis do we determine whether a form of animal use is acceptable or not? How can we justify different treatment of different species or groups of animals? What limits should be placed on animal use? Do animals have rights? Should veterinary team members take/promote a position on animal use? Is it better for veterinary team members to opt out of poor animal welfare (AW) practices or work for change from within settings where AW is poor?	39
Animal welfare (AW) governance	How should AW be legislated, policed, or otherwise protected and promoted? How do agencies charged with enforcement manage conflicts of interest? How is AW governance funded? Is enforcement adequate? Is “ag-gag” legislation acceptable? How should AW legislation, guidelines and policies be interpreted? Under what circumstances should veterinary team members challenge legislation, guidelines, and policies around animal welfare? Should AW be dictated by consumer preference? How should non-stun slaughter be regulated?	30
What should veterinary team members do when clients breach welfare laws or regulations?	Whether to report clients, suspected animal abuse, animal neglect and animal hoarding, animal doping or animal fighting? Should reporting of animal neglect or cruelty be mandatory? How should the veterinary team approach a vulnerable or mentally unwell client who is neglectful of or cruel to animals? Can veterinary team members be compelled not to report clients?	29
Euthanasia of companion animals	What are acceptable grounds for euthanasia? What if consent for euthanasia is contested between owners? To what length should veterinary team members go to establish ownership prior to euthanasia? Which methods of euthanasia are appropriate? How should veterinary team members manage objectionable requests for euthanasia?	28
Research and education	In what circumstances should animals be used in research and education? What limits should be placed on animal use? How should veterinary students be selected? Should universities be influenced by the needs or preferences of animal industries, or the veterinary profession? How should relationships between educational institutions and industry be managed?	28
Ensuring food safety, food security, and biosecurity	How do veterinarians manage conflicts between AW and food safety or food security? How do veterinarians manage conflict between food safety requirements and their client’s productivity? In what circumstances should veterinarians become whistle blowers regarding food safety? How should veterinarians assess and manage risks to food safety? To what extent can food animals be treated for certain conditions? Should food safety controls apply to production animals kept as companions?	27
Scope of practice	What falls within and beyond a veterinarian, veterinary nurse, or animal health technician’s scope of practice? In what circumstances is it acceptable to perform a procedure that is beyond one’s scope of practice? At what point should one refer or defer to an experienced colleague? What if clients pressure veterinary team members to do something beyond their scope of practice? To what extent do responsibilities extend after hours?	26
Confidentiality and privacy	How should veterinary team members manage conflicts between client requests for privacy and AW, public health, or codes of professional conduct? What should veterinary team members do if a one client (e.g., who sells an animal or herd) fails to disclose health information to another client (e.g., the purchaser)? To what extent should veterinary team members respect human privacy? What if the mental wellbeing of people is at stake?	25
Management of errors and complications	When and how should errors be disclosed? How should errors made by other veterinary team members (including those in other practices) be managed? How should veterinary team members be held accountable for errors? What reparations, if any, should be made and what limits, if any, should be placed on these?	25
Conflict of interest (COI)	What counts as a real or perceived COI? Are overservicing and overtreatment due to COI? How should COIs be managed or eliminated?	23
Conflict between the interests of animals and the interests of their owners	Is it reasonable to delay euthanasia of a suffering animal due to client emotional needs? How should the veterinary team member respond if a client can only afford animal treatment by forgoing their own needs? How should animals behaving aggressively, or those that have attacked or injured humans, be treated?	21
How to balance animal productivity with animal welfare	How do veterinary team members manage conflict between productivity and performance (of animals, businesses, or both) with AW? To what extent is it reasonable for an animal or animals to have compromised welfare if they can continue to be productive? How do we assess financial costs associated with improving AW? In what circumstances is it reasonable to transport sick or injured animals?	21
Labelling and use of pharmaceuticals including antimicrobials	What, if any, limits should be placed on use of antimicrobials in animals? How should veterinary team members balance the needs of individual animals and other stakeholders when prescribing or dispensing antimicrobials? What, if any, limits should be placed on drug or prescription diet sales? What factors should be taken into account when considering off-label use or compounding of medications for animals? Are cost concerns justification enough for off-label use of medication?	21
Clients with limited finances	How should the veterinary team proceed if the client does not have immediate funds to provide the recommended treatment? Is it acceptable to provide a lower standard of care where client finances are limited? Under what circumstances is “economic euthanasia” acceptable? Is it acceptable to amend records so that insurers or other third parties cover costs?	20
Collegial relations and wellbeing of veterinary team members	How should conflict between veterinary team members be managed? How should these issues be dealt with in job interviews? What counts as discrimination, bullying or sexual harassment and how should these be addressed? How can veterinary team members manage conflict between personal wellbeing and professional role and maintain appropriate boundaries? How should veterinary team members manage conflicts between loyalty to colleagues and honesty?	19
Working with or assisting other team members who are providing incompetent care	What should veterinary team members do if colleagues, including superiors, provide incompetent care, or care below the acceptable standard of care? What if those colleagues are suffering from health problems, including substance abuse?	19
Shared decision making and informed consent	Under what circumstances is it reasonable to perform a procedure without owner consent? How far can one proceed without consent? Is it ever ethically acceptable to withhold information from a client or clients? What constitutes shared decision making? To what extent is it acceptable, if ever, for a veterinary team member to influence a client? How should veterinary team members manage disagreement regarding consent between different owners of the same animal or animals?	17
Slaughter and killing of farm animals	What methods of slaughter or killing should be used? Which animals should be slaughtered in an emergency animal disease outbreak? Is it acceptable to vary slaughter methods in some situations (e.g., emergency animal disease outbreaks)? Should animals that are surplus to need be slaughtered/humanely killed? Are there viable alternative options?	14
Incorporating evidence into practice and making clinical decisions in the absence of evidence	What constitutes appropriate and acceptable evidence? How should veterinary team members utilise evidence? How should clinical decisions be made where there is scant available evidence, where policies are non-existent or unclear, or where we have a lack of experience? How should we balance published evidence and experience?	13
Management of stray or unowned dogs and cats	Who is responsible for the care and welfare of stray or unowned animals, including costs? Is there a basis for treating stray or unowned animals differently than owned animals? To what extent can veterinary team members police animal ownership? How should the fate of stray or unowned animals be decided?	13
Standard of care (SOC)	What is an appropriate SOC? What about requests to treat below a SOC? What is too high a SOC? What do you do if someone is not providing a minimum SOC? How do you manage variation of SOC across jurisdictions?	11
Treatment and management of wild and free roaming animals	How should we treat individual wildlife patients versus populations? Should wildlife or pest species be treated differently than companion animals? Are particular methods of killing species deemed to be pests ethically acceptable? Can wild or free roaming animals enjoy acceptable welfare?	11
Breeding animals and selecting for particular traits	Is it acceptable to select animals that are better adapted to existing husbandry systems, rather than changing animal husbandry? How can veterinary team members address poor breeding practices whilst ensuring welfare of individual animals? To what extent should human preference inform selection and breeding of animals?	8
Convenience surgeries and mutilations	Are there circumstances in which procedures such as ear cropping, tail docking, debarking, or declawing can be justified? What if colleagues perform these procedures, or clients threaten to perform such procedures themselves?	8
Competition between veterinarians and practices	How to respond to clients from competing practices? Under what circumstances should one report a competing veterinarian or practice for misconduct? Are non-competition causes in contracts acceptable? What limits if any should be placed on these?	8
Futile or non-beneficial treatment of animal patients	At what point is treatment considered futile? How do veterinary team members manage differences of opinion about what treatment is considered futile or non-beneficial? Is it ethical to offer or provide futile or non-beneficial treatment? How and where do veterinary team members draw the line between potentially beneficial and futile treatment?	8
Remuneration and charging for veterinary services and product sales	How should veterinary team members be paid (e.g., salary, performance)? How do practices balance AW with making a profit? How should veterinary products be priced and sold? Is it just to sell products through veterinary channels only?	8
Assessment and measurement of animal welfare and quality of life	How do we resolve differences in animal welfare assessment? How do we ensure that animal welfare and quality of life assessment yield meaningful information?	4

## Data Availability

Not applicable.

## Supplementary Materials

The following are available online at https://www.mdpi.com/article/10.3390/vetsci9010002/s1, S1: Reference list of vignettes analysed in our article.
