# Supplementary material for: What Would You Do? Types of Ethical Challenging Situations Depicted in Vignettes Published in the Veterinary Literature from 1990 to 2020
Source: Vet Sci. 2021 Dec 22;9(1):2. doi: 10.3390/vetsci9010002 (PMC8781959; doi:10.3390/vetsci9010002)
Supplement: Supplementary file 1 [file vetsci-09-00002-s001.zip › vetsci-1484870-supplementary.pdf]

Supplementary material: Reference list of vignettes analysed in our article.

The vignettes analysed in this article were extracted from the following sources: Web of Science (all databases: CAB Abstracts, Current Contents Connect, BIOSIS Previews and MEDLINE), PubMed, Google Scholar and the University of Sydney Library. Not all articles had a Digital Object Identifier (DOI), however where possible we manually added the DOI or alternate identifier (such as PubMed Central number (PMC); PubMed Central Identification (PMCID); PMID (PubMed Identification); Web of Science (WOS) number; or in the case of books an International Standard Book Number (ISBN).

ABBITT, G. 2010. Legal, ethical and professional issues for veterinary nurses. *The Veterinary Nurse,* 1**,** 186-188. https://doi.org/10.12968/vetn.2010.1.3.186

AGLA, S. & ROLLIN, B. E. 2000. An ethicist's commentary on the veterinarian who asks his employee to lie about a surgical death. *The Canadian Veterinary Journal,* 41**,** 520-521. PMCID: PMC1476184

ALAVI, T. 2009. Everyday ethics: seeing malpractice? *In Practice,* 31**,** 250. https://doi.org/10.1136/inpract.31.5.250

ANDERSON, N. & ROLLIN, B. E. 1992. Veterinary medical ethics. *The Canadian Veterinary Journal = La revue veterinaire canadienne,* 33**,** 629-630. 17424085

ANDERSON, N. & ROLLIN, B. E. 2005. Veterinary Medical Ethics Ethical Question of the Month March 2005. *The Canadian Veterinary Journal,* 46**,** 483-483. PMC3085349

ANDERTON, L. & ROLLIN, B. E. 2005. Veterinary Medical Ethics Ethical Question of the Month January 2005. *The Canadian Veterinary Journal,* 46**,** 299-299. PMC3085339

ANZUINO, K. 2007. Everyday ethics: conflicting co-owners. *In Practice,* 29**,** 234-234. https://doi.org/10.1136/inpract.29.4.234

ANZUINO, K. 2010. Everyday ethics: disbudding illegally. *In Practice,* 32**,** 78. https://doi.org/10.1136/inpract.32.2.78

APPELT, M. & ROLLIN, B. E. 2012. Veterinary Medical Ethics Ethical Question of the Month May 2012. *The Canadian Veterinary Journal,* 53**,** 465-468. PMC3327584

ASHALL, V. 2009. Everyday ethics: canine blood donor. *In Practice,* 31**,** 527-527. https://doi.org/10.1136/inpract.31.10.527

ASHALL, V. 2014. Everyday ethics: Euthanasia in a no-kill shelter. *In Practice,* 36**,** 478. https://doi.org/10.1136/inp.g5723

ATKINSON, M. 2006. Everyday ethics: Difficult Dog. *In Practice,* 28**,** 559. https://doi.org/10.1136/inpract.28.9.559

ATKINSON, M. 2007. Everyday ethics: maliciously injured kitten. *In Practice,* 29**,** 422-422. https://doi.org/10.1136/inpract.29.7.422

ATKINSON, M. 2008. Everyday ethics: a patient abroad. *In Practice,* 30**,** 352-353. https://doi.org/10.1136/inpract.30.6.352

AU, C. 2020. Dealing with the client who wishes to take their hyperthyroid ferret home to die naturally. *In Practice,* 42**,** 309. https://doi.org/10.1136/inp.m1967

AWAD, M. & STEPHENS, T. 2016. What to do if a client asks you to change your records for a claim. *Australian Veterinary Journal,* 94**,** N22-23.

BARKER, C. 2010. Everyday ethics: can't pay, won't pay. *In Practice,* 32**,** 413-414. https://doi.org/10.1136/inpract.32.8.413

BARTON, F. & ROLLIN, B. 1997. An ethicist's commentary on the case of the bull mastiff with osteosarcoma. *The Canadian Veterinary Journal = La revue veterinaire canadienne,* 38**,** 536-537. 9285134

BAZELEY, K. 2007. Everyday ethics: charitable aspirations. *In Practice,* 29**,** 176. https://doi.org/10.1136/inpract.29.3.176

BERNARD, M. A. & ROLLIN, B. 1993. Veterinary medical ethics. *The Canadian Veterinary Journal = La revue veterinaire canadienne,* 34**,** 519-520. 17424280

BILL, R. 2016. Convenience Euthanasia. *In:* TRAN, C. V., DESANTIS KERR, A., BILL, R. & WALSH, J. S. (eds.) *Exploring the Gray Zone.* Purdue University Press. https://doi.org/10.2307/j.ctt163t7qs.8

BILL, R. 2016. Popular Party Guy or Ethical Concern? *In:* TRAN, C. V., DESANTIS KERR, A., BILL, R. & WALSH, J. S. (eds.) *Exploring the Gray Zone.* Purdue University Press. https://doi.org/10.2307/j.ctt163t7qs.19

BILL, R. 2016. She Said What? Gossip in the Workplace. *In:* TRAN, C. V., DESANTIS KERR, A., BILL, R. & WALSH, J. S. (eds.) *Exploring the Gray Zone.* Purdue University Press. https://doi.org/10.2307/j.ctt163t7qs.13

BILL, R. 2016. When Roles of Veterinarians and Veterinary Technicians Blur. *In:* TRAN, C. V., DESANTIS KERR, A., BILL, R. & WALSH, J. S. (eds.) *Exploring the Gray Zone.* Purdue University Press. https://doi.org/10.2307/j.ctt163t7qs.16

BONNETT, B. & ROLLIN, B. E. 1992. Veterinary medical ethics. *The Canadian Veterinary Journal = La revue veterinaire canadienne,* 33**,** 220-221. PMID: 17423979

BROWN, R. 2015. Everyday ethics: Accounting for a lame cow. *In Practice,* 37**,** 485. https://doi.org/10.1136/inp.h5012

BROWN, R. 2015. Everyday ethics: Billy the kid. *In Practice,* 37**,** 374. https://doi.org/10.1136/inp.h2818

BROWN, R. 2015. Everyday ethics: Delivering profit? *In Practice,* 37**,** 550. https://doi.org/10.1136/inp.h5855

BROWN, R. 2015. Everyday ethics: Immediate dispatch. *In Practice,* 37**,** 262. https://doi.org/10.1136/inp.h2274

BROWN, R. 2015. Everyday ethics: Possession is nine-tenths of the dog. *In Practice,* 37**,** 310. https://doi.org/10.1136/inp.h3223

BROWN, R. 2016. Everyday ethics: Questioning morals. *In Practice,* 38**,** 150. https://doi.org/10.1136/inp.i863

BROWN, R. 2016. Everyday ethics: When in Rome. *In Practice,* 38**,** 46. https://doi.org/10.1136/inp.h6401

BROWN, R. 2017. Everyday ethics: Witnessing farm animal abuse during work experience. *In Practice,* 39**,** 342. https://doi.org/10.1136/inp.j3282

BRUGMAN, A. 2020. Dealing with a case of suspected 'petfishing'. *In Practice,* 42**,** 589. https://doi.org/10.1136/inp.m4473

BUTTERWORTH, A. & MULLAN, S. 2006. Everyday ethics - Cetacean stranding. *In Practice,* 28**,** 499-499. https://doi.org/10.1136/inpract.28.8.499

CAMPBELL, M. 2016. Everyday ethics: Too hot to handle? *In Practice,* 38**,** 414. https://doi.org/10.1136/inp.i4468

CANNON, M. 2011. Everyday ethics: Farm cat with TB. *In Practice,* 33**,** 46. https://doi.org/10.1136/inp.c7331

CASEY, R. 2011. Everyday ethics: mammary mass in an overweight dog. *In Practice,* 33**,** 493-494. https://doi.org/10.1136/inp.d5746

CEELEN, H. & ROLLIN, B. E. 1998. An ethicist's commentary on maintaining confidentiality in the face of a previous client selling sick animals. *The Canadian Veterinary Journal = La revue veterinaire canadienne,* 39**,** 72-73. 10051954

CHEUNG, W. & FAWCETT, A. 2019. Managing a high publicity crowdfunded shelter puppy with head trauma. *In Practice,* 41**,** 182-3. https://doi.org/10.1136/inp.l1889

CHEUNG, W. & FAWCETT, A. 2019. Rehoming a surrendered dog to its original owner. *In Practice,* 41**,** 278-278. https://doi.org/10.1136/inp.l4489

CHOW, L. & ROLLIN, B. E. 2016. Veterinary Medical Ethics Ethical question of the month — November 2016. *The Canadian Veterinary Journal,* 57**,** 1123-1126. PMC5081141

COGHLAN, S. 2017. Everyday ethics: Euthanasing treatable patients. *In Practice,* 39**,** 190. https://doi.org/10.1136/inp.j1256

COGHLAN, S. 2017. Everyday ethics: When the client is away. *In Practice,* 39**,** 94-95. https://doi.org/10.1136/inp.j371

COGHLAN, S. 2018. Everyday ethics: Dealing with dogs that bite. *In Practice,* 40**,** 470. https://doi.org/10.1136/inp.k4938

COGHLAN, S. 2018. Everyday ethics: Legal status and malpractice. In Practice, 40, 78. https://doi.org/10.1136/inp.k823

COGHLAN, S. 2019. Enrolling in animal-assisted therapy programmes. *In Practice,* 41**,** 134. https://doi.org/10.1136/inp.l1611

COGHLAN, S. & CARDILINI, A. 2019. When your views on climate change conflict with your chosen career path. *In Practice,* 41**,** 461. https://doi.org/10.1136/inp.l6101

COLES, G. 2010. Everyday ethics: advice on horse worming. *In Practice,* 32**,** 170-170. https://doi.org/10.1136/inp.c1532

COLLINS, J. 2014. Everyday ethics: Looking a store horse in the mouth. *In Practice,* 36**,** 158. https://doi.org/10.1136/inp.g1426

COOMBS, C. & ROLLIN, B. E. 1996. An ethicist's commentary on the case of whether a veterinarian is obliged to relate a previous employee's history of drug abuse to a prospective employer. *The Canadian Veterinary Journal = La revue veterinaire canadienne,* 37**,** 456-457. 8853879

COUGHLIN, B. & ROLLIN, B. E. 1998. An ethicist's commentary on the case of the cat who fractures both legs after a declaw. *The Canadian Veterinary Journal = La revue veterinaire canadienne,* 39**,** 337-338. 9635169

COUSQUER, G. 2011. Everyday ethics: pet insurance problem. *In Practice,* 33**,** 237-238. https://doi.org/10.1136/inp.d2287

COUSQUER, G. 2011. Everyday ethics: Principled profit-sharing? *In Practice,* 33**,** 142. https://doi.org/10.1136/inp.d957

COUSQUER, G. 2012. Everyday ethics: Grey squirrel treatment and hand-rearing. *In Practice,* 34**,** 550-551. https://doi.org/10.1136/inp.e6272

COUSQUER, G. 2013. Everyday ethics: Being a badger's advocate. *In Practice,* 35**,** 350-U58. https://doi.org/10.1136/inp.f3383

COUSQUER, G. 2015. Everyday ethics: Emergency exsanguination of working equids. *In Practice,* 37**,** 102-U38. https://doi.org/10.1136/inp.h96

CRANLEY, J. 2017. Everyday ethics: Ethical issues in the slaughterhouse. *In Practice,* 39**,** 430. https://doi.org/10.1136/inp.j4497

CULJAT-VUKMAN, E. 2020. Reporting a case of suspected dog fighting. *In Practice,* 42**,** 365. https://doi.org/10.1136/inp.m2393

CVJ 1990. Veterinary medical ethics. *The Canadian Veterinary Journal = La revue veterinaire Canadienne, 31,* 739-739

CVJ AND ROLLIN, B. E. 2007. Veterinary Medical Ethics Ethical Question of the Month August 2007. *The Canadian Veterinary Journal,* 48**,** 791-792. PMC1914309

CVJ AND ROLLIN, B. E. 2007. Veterinary Medical Ethics Ethical Question of the Month July 2007. *The Canadian Veterinary Journal,* 48**,** 679-682. PMC1899841

CVJ AND ROLLIN, B. E. 2007. Veterinary Medical Ethics Ethical Question of the Month September 2007. *The Canadian Veterinary Journal,* 48**,** 895-898. PMC1950106

DE SANTIS KERR, A. 2016. How Much Help Is Too Much Help? *In:* DESANTIS KERR, A., BILL, R., X201C, PETE, X201D, WALSH, J. S. & TRAN, C. V. (eds.) *Exploring the Gray Zone.* Purdue University Press. https://doi.org/10.2307/j.ctt163t7qs.6

DELANEY, K. & ROLLIN, B. E. 1997. An ethicist's commentary on the case of the infected research animal. *The Canadian Veterinary Journal = La revue veterinaire canadienne,* 38**,** 136-136. 9056065

DENNISON, T. 2009. Everyday ethics: dog needs a new home. *In Practice,* 31**,** 147-147. https://doi.org/10.1136/inpract.31.3.147

DENNISON, T. & MULLAN, S. 2009. Everyday ethics: vaccination with a twitch. *In Practice,* 31**,** 302-303. https://doi.org/10.1136/inpract.31.6.302

DESANTIS KERR, A. 2016. Between a Rock and a Hard Place. *In:* TRAN, C. V., DESANTIS KERR, A., BILL, R. & WALSH, J. S. (eds.) *Exploring the Gray Zone.* Purdue University Press. https://doi.org/10.2307/j.ctt163t7qs.10

DESANTIS KERR, A. 2016. Compliment? Or Reason for Concern? *In:* TRAN, C. V., DESANTIS KERR, A., BILL, R. & WALSH, J. S. (eds.) *Exploring the Gray Zone.* Purdue University Press. https://doi.org/10.2307/j.ctt163t7qs.30

DESANTIS KERR, A. 2016. Contagious Disease and Confidentiality. *In:* TRAN, C. V., DESANTIS KERR, A., BILL, R. & WALSH, J. S. (eds.) *Exploring the Gray Zone.* Purdue University Press. https://doi.org/10.2307/j.ctt163t7qs.15

DESANTIS KERR, A. 2016. The Helpful Neighbor or the Meddling Neighbor? *In:* TRAN, C. V., DESANTIS KERR, A., BILL, R. & WALSH, J. S. (eds.) *Exploring the Gray Zone.* Purdue University Press. https://doi.org/10.2307/j.ctt163t7qs.31

DESANTIS KERR, A. 2016. On Call Dilemma. *In:* TRAN, C. V., DESANTIS KERR, A., BILL, R. & WALSH, J. S. (eds.) *Exploring the Gray Zone.* Purdue University Press. https://doi.org/10.2307/j.ctt163t7qs.28

DESANTIS KERR, A. 2016. Standard of Care? *In:* TRAN, C. V., DESANTIS KERR, A., BILL, R. & WALSH, J. S. (eds.) *Exploring the Gray Zone.* Purdue University Press. https://doi.org/10.2307/j.ctt163t7qs.17

DESANTIS KERR, A. 2016. Suspicious Client? *In:* TRAN, C. V., DESANTIS KERR, A., BILL, R. & WALSH, J. S. (eds.) *Exploring the Gray Zone.* Purdue University Press. https://doi.org/10.2307/j.ctt163t7qs.23

DESANTIS KERR, A. 2016. What about the Lambs? *In:* TRAN, C. V., DESANTIS KERR, A., BILL, R. & WALSH, J. S. (eds.) *Exploring the Gray Zone.* Purdue University Press. https://doi.org/10.2307/j.ctt163t7qs.12

DESANTIS KERR, A. 2016. What's in an answer? *In:* TRAN, C. V., DESANTIS KERR, A., BILL, R. & WALSH, J. S. (eds.) *Exploring the Gray Zone.* Purdue University Press. https://doi.org/10.2307/j.ctt163t7qs.18

DILLMAN, E. & ROLLIN, B. E. 2006. Ethical Question of the Month November 2005. *The Canadian Veterinary Journal,* 47**,** 109-110. PMC3085498

DIXON, G. 2006. Everyday ethics: Gets Your Goat. *In Practice,* 28**,** 298-299. https://doi.org/10.1136/inpract.28.5.298

DIXON, G. 2008. Everyday ethics: shooting for pleasure. *In Practice,* 30**,** 584. https://doi.org/10.1136/inpract.30.10.584

DUBOIS BISSETT, A. & ROLLIN, B. E. 1994. An ethicist's commentary on the case of a referral practice attempting to "steal" clients. *The Canadian Veterinary Journal = La revue veterinaire canadienne,* 35**,** 262-262. 8050071

DUFFIELD, T. & ROLLIN, B. E. 2015. Veterinary Medical Ethics Ethical question of the month — January 2015. *The Canadian Veterinary Journal,* 56**,** 15-17. PMC4266050

DYKES, L. 2008. Everyday ethics: rabbit tooth abscess. *In Practice,* 30**,** 232. https://doi.org/10.1136/inpract.30.4.232

ENTICOTT, G. 2010. Everyday ethics: TB testing protocol. *In Practice,* 32**,** 318-319. https://doi.org/10.1136/inp.c3677

EVANS, E. 2020. Performing emergency care without client consent. *In Practice,* 42**,** 181. https://doi.org/10.1136/inp.m1144

FAFARD, A. & ROLLIN, B. E. 2012. Veterinary Medical Ethics Ethical Question of the Month July 2012. *The Canadian Veterinary Journal,* 53**,** 705-707. PMC3377454

FAWCETT, A. 2009. Everyday ethics: how much information? *In Practice,* 31**,** 409-409. https://doi.org/10.1136/inpract.31.8.409

FAWCETT, A. 2010. Everyday ethics: Insulin overdose. *In Practice,* 32**,** 270. https://doi.org/10.1136/inp.c2931

FAWCETT, A. 2010. Everyday ethics: the wrong vaccine. *In Practice,* 32**,** 214-215. https://doi.org/10.1136/inpract.32.4.214

FAWCETT, A. 2013. Everyday ethics: Anaesthetic death: who pays? *In Practice,* 35**,** 286-U83. https://doi.org/10.1136/inp.f2556

FAWCETT, A. 2017. Everyday ethics: Brachycephalic dogs and honesty with clients. *In Practice,* 39**,** 45. https://doi.org/10.1136/inp.i6329

FAWCETT, A. 2018. Everyday ethics: One health and antimicrobial resistance. *In Practice,* 40**,** 126-126. https://doi.org/10.1136/inp.k1442

FAWCETT, A. 2019. Feeding cats a plant-based diet. *In Practice,* 41**,** 341. https://doi.org/10.1136/inp.l4950

FAWCETT, A. & BAGULEY, J. 2011. Everyday ethics: Social media menace? *In Practice,* 33**,** 190. https://doi.org/10.1136/inp.d1698

FAWCETT, A. & BRAILEY, J. 2012. Everyday ethics: Are you positive? the fate of a shelter cat. *In Practice,* 34**,** 614- https://doi.org/10.1136/inp.e6710

FAWCETT, A. & BRAILEY, J. 2017. Everyday ethics: Taking a full lunch break. *In Practice,* 39**,** 238-239. https://doi.org/10.1136/inp.j1971

FAWCETT, A. & CHADWICK, M. 2018. Everyday ethics: Clients who cannot afford to pay. *In Practice,* 40**,** 38. https://doi.org/10.1136/inp.k118

FAWCETT, A. & GOTTLIEB, T. 2017. Everyday ethics: Performance indicators and prescribing antimicrobials. *In Practice,* 39**,** 382. https://doi.org/10.1136/inp.j4004

FAWCETT, A. & WOON, S. 2013. Everyday ethics: Pest or patient? *In Practice,* 35**,** 614. https://doi.org/10.1136/inp.f6114

FLEMMING, B. I. & ROLLIN, B. E. 2000. An ethicist's commentary on the case of client who won't euthanize a suffering foal. *The Canadian Veterinary Journal,* 41**,** 830-831. PMC1476429

FOELL, C. & ROLLIN, B. E. 1997. Ethicist's second commentary on the case of the stray tattooed beagle. *The Canadian Veterinary Journal = La revue veterinaire canadienne,* 38**,** 472-473. 9262855

FORDYCE, P. 2011. Everyday ethics: violent vet. *In Practice,* 33**,** 94-95. https://doi.org/10.1136/inp.d297

FORDYCE, P. 2013. Everyday ethics: Honesty and euthanasia. *In Practice,* 35**,** 422. https://doi.org/10.1136/inp.f4458

FORDYCE, P. 2017. Everyday ethics: When the client cannot pay. *In Practice,* 39**,** 142-U80. https://doi.org/10.1136/inp.j905

FORREST, N. 2008. Everyday ethics: a question of ownership. *In Practice,* 30**,** 292. https://doi.org/10.1136/inpract.30.5.292

FORREST, N. 2008. Everyday ethics: veterinary nurse uneasy with vet's approach. *In Practice,* 30**,** 292. https://doi.org/10.1136/inpract.29.8.489

GARDINER, A. 2007. Everyday ethics: has small animal practice gone too far. *In Practice,* 29**,** 553-553. https://doi.org/10.1136/inpract.29.9.553

GARTLEY, C. & ROLLIN, B. E. 1991. Veterinary medical ethics. *The Canadian Veterinary Journal = La revue veterinaire canadienne,* 32**,** 390-391. 17423815

GERROW, T. & ROLLIN, B. E. 1994. An ethicist's commentary on the case of the improperly labelled prescription. *The Canadian Veterinary Journal = La revue veterinaire canadienne,* 35**,** 202-203. 8076275

GODKIN, A. & ROLLIN, B. 1993. Veterinary medical ethics. *The Canadian Veterinary Journal = La revue veterinaire canadienne,* 34**,** 72-73. 17424174

GODKIN, A. & ROLLIN, B. E. 2015. Veterinary Medical Ethics Ethical question of the month — February 2015. *The Canadian Veterinary Journal,* 56**,** 121-122. PMC4298261

GODKIN, A. & ROLLIN, B. E. 2017. Veterinary Medical Ethics Ethical question of the month - August 2017. *The Canadian Veterinary Journal = La revue veterinaire canadienne,* 58**,** 781-783. 28761182

GOEREE, G. & ROLLIN, B. E. 2001. An ethicist's commentary on veterinary involvement with laws banning pitbulls and pitbull crosses. *The Canadian Veterinary Journal = La revue veterinaire canadienne,* 42**,** 258-259. 11326629

GOEREE, G. & ROLLIN, B. E. 2008. Veterinary Medical Ethics Ethical Question of the Month January 2008. *The Canadian Veterinary Journal,* 49**,** 15-16. PMC2147688

GOEREE, G. & ROLLIN, B. E. 2008. Veterinary Medical Ethics Ethical Question of the Month September 2008. *The Canadian Veterinary Journal,* 49**,** 845-846. PMC2519904

GREEN, R. 2007. Everyday ethics: charity's stance on hereditary defects. *In Practice,* 29**,** 624. https://doi.org/10.1136/inpract.29.10.624

GREEN, R. 2012. Everyday ethics: shortcomings in locum practice procedures. *In Practice,* 34**,** 110-111. https://doi.org/10.1136/inp.e71

GREEN, R. 2014. Everyday ethics: Follow-up fiasco. *In Practice,* 36**,** 374. https://doi.org/10.1136/inp.g4255

GREY-BRUCE VETERINARY ASSOCIATION & ROLLIN, B. E. 2007. Veterinary Medical Ethics Ethical Question of the Month January 2007. *The Canadian Veterinary Journal,* 48**,** 17-18. PMC1716747

HACKMAN, N. 2004. A Question of Conscience: Assistants and Anaesthesia. *Veterinary Technician,* 25**,** 714-715.

HACKMAN, N. 2005. A question of conscience - Client needs versus practice profits. *Veterinary Technician,* 26**,** 806. WOS:000233471400013.

HARPER, M. & ROLLIN, B. E. 2017. Veterinary Medical Ethics - Ethical Question of the Month December 2017. *The Canadian Veterinary Journal = La revue veterinaire canadienne,* 58**,** 1253-1255. 29203934

HASLAM, S. 2006. Everyday ethics: In the owner's interests. *In Practice,* 28**,** 159-159. https://doi.org/10.1136/inpract.28.3.159

HASLAM, S. 2008. Everyday ethics: who owns a stray dog? In Practice, 30, 529. https://doi.org/10.1136/inpract.30.9.529

HELE, E. 2018. Everyday ethics: Pleural effusion in a geriatric cat. *In Practice,* 40**,** 310. https://doi.org/10.1136/inp.k3650

HENDERSON, K. 2013. Everyday ethics: Confronting bad husbandry. *In Practice,* 35**,** 486. https://doi.org/10.1136/inp.f4716

HENDERSON, K. 2019. Disclosing pre-existing veterinary conditions to insurance companies. *In Practice,* 41**,** 46-47. https://doi.org/10.1136/inp.l125

HERBERT, R. & ROLLIN, B. E. 2005. Veterinary Medical Ethics Ethical Question of the Month September 2005. *The Canadian Veterinary Journal,* 46**,** 1081-1082. PMC3085390

HOLOWAYCHUK, M. & ROLLIN, B. E. 2008. Veterinary Medical Ethics Ethical Question of the Month May 2008. *The Canadian Veterinary Journal,* 49**,** 439-440. PMC2359487

HUNTLEY, E. 2019. When your duty of care extends beyond the patient to the client. *In Practice,* 41**,** 517. https://doi.org/10.1136/inp.l6475

INGWERSEN, W. & ROLLIN, B. E. 1999. Veterinary Medical Ethics. *The Canadian Veterinary Journal,* 40**,** 846-847. PMC1539874

JOSEPHSON, G. & ROLLIN, B. E. 1995. An ethicist's commentary on the case of a veterinarian with a client who has illicitly imported boar semen. *The Canadian Veterinary Journal = La revue veterinaire canadienne,* 36**,** 674-674. 8590422

KEELER, K. & ROLLIN, B. E. 1994. An ethicist's commentary on the found dog cases. *The Canadian Veterinary Journal = La revue veterinaire canadienne,* 35**,** 537-537. 7994716

KERR, A. 2008. Everyday ethics: pregnant downer cow. *In Practice,* 30**,** 117 https://doi.org/10.1136/inpract.30.2.117 .

KERR, A. 2009. Everyday ethics: injured fawn. *In Practice,* 31**,** 470-471. https://doi.org/10.1136/inpract.31.9.470

KERR, A. 2009. Everyday ethics: Suspected Organised Dog Fight. *In* Practice*,* 31**,** 198. https://doi.org/10.1136/inpract.31.4.198

KLUMPP L, B. R. 2010. Chimpanzee scenario. *Australian Veterinary Journal,* 88**,** N14-15.

KNIGHT, A. 2014. Everyday ethics: Dealing with dark desires. *In Practice,* 36**,** 54. https://doi.org/10.1136/inp.g375

KNIGHT, A. 2016. Everyday ethics: A request for euthanasia: advising a colleague. *In Practice,* 38**,** 469. https://doi.org/10.1136/inp.i5051

KNIGHT, A. 2016. Everyday ethics: A request for euthanasia: handling the client. *In Practice,* 38**,** 358. https://doi.org/10.1136/inp.i3672

KNIGHT, A. 2017. Everyday ethics: Advice requested via social media. *In Practice,* 39**,** 478. https://doi.org/10.1136/inp.j5005

KWANTES, L. & ROLLIN, B. 1993. Veterinary medical ethics. *The Canadian Veterinary Journal = La revue veterinaire canadienne,* 34**,** 201-202. 17424197

LANGLAIS, L. & ROLLIN, B. E. 2005. Veterinary Medical Ethics Ethical Question of the Month February 2005. *The Canadian Veterinary Journal,* 46**,** 395-395. PMC3085343

LEE, J., WELSH, P. & WHITING, M. 2013. Professional and legal issues: surgical misadventure. *The Veterinary Nurse,* 4**,** 504-507. https://doi.org/10.12968/vetn.2013.4.8.504

LEEB, C. 2007. Everyday ethics: a troubled vet abroad. *In Practice,* 29**,** 304-305. https://doi.org/10.1136/inpract.29.5.304

LEM, M. & ROLLIN, B. E. 2012. Veterinary Medical Ethics Ethical Question of the Month June 2012. *The Canadian Veterinary Journal,* 53**,** 591-594. PMC3354817

LEWIS, R. & ROLLIN, B. E. 2009. Veterinary Medical Ethics Ethical Question of the Month April 2009. *The Canadian Veterinary Journal,* 50**,** 345-349. PMC2657514

LEWIS, R. & ROLLIN, B. E. 2009. Veterinary Medical Ethics Ethical Question of the Month May 2009. *The Canadian Veterinary Journal,* 50**,** 455-459. PMC2671867

LEWIS, R. & ROLLIN, B. E. 2010. Veterinary Medical Ethics Ethical Question of the Month August 2010. *The Canadian Veterinary Journal,* 51**,** 807-810. PMC2904998

LEWIS, R. & ROLLIN, B. E. 2011. Veterinary Medical Ethics Ethical Question of the Month July 2011. *The Canadian Veterinary Journal,* 52**,** 701-704. PMC3119234

LEWIS, R. & ROLLIN, B. E. 2012. Veterinary Medical Ethics Ethical Question of the Month October 2012. *The Canadian Veterinary Journal,* 53**,** 1031-1034. PMC3447306

LEWIS, R. & ROLLIN, B. E. 2018. Veterinary Medical Ethics - Ethical Question of the Month June 2018. *The Canadian Veterinary Journal = La revue veterinaire canadienne,* 59**,** 577-579. 29910472

LEWIS, R. & ROLLIN, B. E. 2018. Veterinary Medical Ethics - Ethical Question of the Month May 2018. *The Canadian Veterinary Journal = La revue veterinaire canadienne,* 59**,** 465-466. 29904199

LULAI, C. & ROLLIN, B. E. 2003. An ethicist's commentary on the elastrator for older bulls. *The Canadian veterinary journal = La revue veterinaire canadienne,* 44**,** 624-624. 13677594

MAASTRICHT, S. 2010. Ethical dilemma - Sheba. *Australian Veterinary Journal,* 88**,** N22-3.

MACDONALD, J. 2011. Who needs key skills? Part 2. *Veterinary Nursing Journal,* 26**,** 18-20. https://doi.org/10.1111/j.2045-0648.2010.00006.x

MACLEOD, A. & ROLLIN, B. E. 2013. Veterinary Medical Ethics Ethical Question of the Month August 2013. *The Canadian Veterinary Journal,* 54**,** 725-727. PMC3711157

MAGALHÃES-SANT'ANA, M. 2016. Everyday ethics: Refusing to take part in euthanasia. *In Practice,* 38**,** 253. https://doi.org/10.1136/inp.i1959

MAGALHÃES-SANT'ANA, M. 2017. Everyday ethics: Resisting the urge to prescribe vancomycin. *In Practice,* 39**,** 294. https://doi.org/10.1136/inp.j2410

MAGALHÃES-SANT'ANA, M. 2020. Sedating a horse for the purpose of film production. *In Practice,* 42**,** 245. https://doi.org/10.1136/inp.m1493

MAGALHÃES-SANT'ANA, M.; HANLON, A.J. Straight from the horse's mouth: Using vignettes to support student learning in veterinary ethics. Journal of Veterinary Medical Education 2016, 43, 321-330. https://doi.org/10.3138/jvme.0815-137R1

MAGALHÃES-SANT'ANA, M., MORE, S. J., MORTON, D. B. & HANLON, A. 2016. Ethical challenges facing veterinary professionals in Ireland: results from Policy Delphi with vignette methodology. Vet Rec, 179, 437. https://doi.org/10.1136/vr.103786

MAGALHÃES, S., ANA, M., PELETEIRO, M. C. & STILWELL, G. 2020. Establishing Animal Welfare Rules of Conduct for the Portuguese Veterinary Profession—Results from a Policy Delphi with Vignettes. Animals, 10. https://doi.org/10.3390/ani10091596

MAIN, C. 2010. Everyday ethics: postoperative responsibility. *In Practice,* 32**,** 38-39. https://doi.org/10.1136/inpract.32.1.38

MAIN, D. 2014. Everyday ethics: Financial incentives. *In Practice,* 36**,** 262. https://doi.org/10.1136/inp.g2826

MALIK, A. 2020. Treating exotics when your colleague has limited expertise. *In Practice,* 42**,** 61. https://doi.org/10.1136/inp.m124

MALIK, A. 2020. What should you do with an orphaned baby grey squirrel? *In Practice,* 42**,** 469. https://doi.org/10.1136/inp.m3250

MCCAMUS, J. & ROLLIN, B. E. 2001. An ethicist's commentary on the case of a client with too many animals. *The Canadian Veterinary Journal = La revue veterinaire canadienne,* 42**,** 853-854. 11708202

MCCAMUS, J. & ROLLIN, B. E. 2002. An ethicist's commentary on the elderly couple rescuing animals. *The Canadian Veterinary Journal,* 43**,** 327-328. PMC339249

MCCULLOCH, S. 2011. Everyday ethics: financially strapped owner with a suffering cat. *In Practice,* 33**,** 297-298. https://doi.org/10.1136/inp.d3465

MCCULLOCH, S. 2012. Everyday ethics: what if it was your dog? *In Practice,* 34**,** 494-495. https://doi.org/10.1136/inp.e5530

MCCULLOCH, S. 2013. Everyday ethics: Is flirting with clients taboo? *In Practice,* 35**,** 222. https://doi.org/10.1136/inp.f2034

MCCULLOCH, S. 2014. Everyday ethics: The case of the whelping bitch. *In Practice,* 36**,** 102. https://doi.org/10.1136/inp.g145

MCKELVEY, D. D. & ROLLIN, B. E. 2006. Veterinary Medical Ethics Ethical Question of the Month July 2006. *The Canadian Veterinary Journal,* 47**,** 631-634. PMC1482447

MCLELLAN, L. 2018. Everyday ethics: Slipped disc and dyspnoea in a French bulldog. *In Practice,* 40**,** 166. https://doi.org/10.1136/inp.k1793

MCMURRAN, S. 2020. Administering an analgesic during electroejaculation procedures in rams. *In Practice,* 42**,** 421. https://doi.org/10.1136/inp.m2645

MCNEIL, M. & ROLLIN, B. E. 2012. Veterinary Medical Ethics Ethical Question of the Month September 2012. *The Canadian Veterinary Journal,* 53**,** 919-922. PMC3418777

MILLS, D. 2016. Everyday ethics: Too feral to save? *In Practice,* 38**,** 198. https://doi.org/10.1136/inp.i1532

MILLS, D. 2016. Everyday ethics: Using the evidence. *In Practice,* 38**,** 310. https://doi.org/10.1136/inp.i3023

MULLAN, S. 2006. Everyday ethics: Celebrity Downer Cow. *In Practice,* 28**,** 52. https://doi.org/10.1136/inpract.28.1.52

MULLAN, S. 2006. Everyday ethics: Homeopathy, a moral stress? *In Practice,* 28**,** 422-422. https://doi.org/10.1136/inpract.28.7.422

NEALE, M. 2007. Everyday ethics: organic farm with languishing lambs. *In Practice,* 29**,** 367-367. https://doi.org/10.1136/inpract.29.6.367

NEALE, M. 2008. Everyday ethics: substandard stockmanship. *In Practice,* 30**,** 416. http://dx.doi.org/10.1136/inpract.30.7.416

NEMETH, N. & ROLLIN, B. E. 2002. An ethicist's commentary on the mishandling of an injured animal by a raptor rehabilitation facility. *The Canadian Veterinary Journal = La revue veterinaire canadienne,* 43**,** 666-667. 12240524

NORTH AMERICAN VETERINARY TECHNICIAN ASSOCIATION 2001. A question of conscience - Suspected abuse. *Veterinary Technician,* 22**,** 557, 561. WOS:000171715900013

NORTH AMERICAN VETERINARY TECHNICIAN ASSOCIATION 2002. A question of conscience: Breeding grounds of trouble. *Veterinary Technician,* 23**,** 239-239. WOS:000175007300010

NORTH AMERICAN VETERINARY TECHNICIAN ASSOCIATION 2003. A question of conscience. *Veterinary Technician,* 24**,** 492-493. WOS:000184418900029

O'HALLORAN, J. & ROLLIN, B. E. 2017. Veterinary Medical Ethics - Ethical Question of the Month November 2017. *The Canadian Veterinary Journal = La revue veterinaire canadienne,* 58**,** 1149-1151. 29089650

OLFERT, E. & ROLLIN, B. E. 1992. Veterinary medical ethics. *The Canadian Veterinary Journal = La revue veterinaire canadienne,* 33**,** 775-777. 17424129

PETRENY, L. & ROLLIN, B. E. 2007. Veterinary Medical Ethics Ethical Question of the Month April 2007. *The Canadian Veterinary Journal,* 48**,** 345-348. PMC1831507

PETRENY, L. & ROLLIN, B. E. 2008. Veterinary Medical Ethics Ethical Question of the Month March 2008. *The Canadian Veterinary Journal,* 49**,** 227-229. PMC2249713

PHIPPS, B. & ROLLIN, B. 1993. Veterinary medical ethics. *The Canadian Veterinary Journal = La revue veterinaire canadienne,* 34**,** 586-587. 17424302

PRITCHARD, J. 2006. Everyday ethics: to trial or not to trial. *In Practice,* 28**,** 359-360. https://doi.org/10.1136/inpract.28.6.359

PRITCHARD, J. 2006. Everyday ethics: Trotter Struggling to Walk. *In Practice,* 28**,** 228-229. https://doi.org/10.1136/inpract.28.4.228

RAMEY, D. & ROLLIN, B. E. 2014. Veterinary Medical Ethics Ethical Question of the Month June 2014. *The Canadian Veterinary Journal,* 55**,** 415-416. PMC3992301

RAMSEY, W. B. & ROLLIN, B. E. 2000. An ethicist's commentary on whether a technician should administer preanesthesia without a veterinary examination. *The Canadian Veterinary Journal,* 41**,** 592-593. PMC1476242

REES, G. 2014. Everyday ethics: Fate of a severely lame cow. *In Practice,* 36**,** 526. https://doi.org/10.1136/inp.g6399

REES, G. 2015. Everyday ethics: Always tell the truth? *In Practice,* 37**,** 150. https://doi.org/10.1136/inp.h971

REES, G. 2015. Everyday ethics: The mating game. *In Practice,* 37**,** 206. https://doi.org/10.1136/inp.h1303

RICHARDS, I. 2013. Everyday ethics: Rodents and rights: patients or pet food? *In Practice,* 35**,** 158. https://doi.org/10.1136/inp.f829

ROBERTS, A. 2020. Dealing with the owner who is reluctant to accept that their pet is obese. *In Practice,* 42**,** 533. https://doi.org/10.1136/inp.m3865

ROBERTS, D. & ROLLIN, B. E. 2003. An ethicist's commentary on veterinarians treating unowned animals and euthanizing unwanted animals. *The Canadian Veterinary Journal = La revue veterinaire canadienne,* 44**,** 363-364. 12757128

ROBINSON, R. A. & ROLLIN, B. E. 1996. An ethicist's commentary on the veterinarian receiving complaints from swine barn workers concerning respiratory problems. *The Canadian Veterinary Journal = La revue veterinaire canadienne,* 37**,** 262-263. 8705970

ROBINSON, R. A. & ROLLIN, B. E. 1997. An ethicist's commentary on the case of the anorexic client who does not feed her dog. *The Canadian Veterinary Journal = La revue veterinaire canadienne,* 38**,** 263-263. 9167875

ROGER, P. 2007. Everyday ethics: neuter or just treat? *In Practice,* 29**,** 55-55. https://doi.org/10.1136/inpract.29.1.55

ROGER, P. 2009. Everyday ethics: bulldog caesarian. *In Practice,* 31**,** 95-95. https://doi.org/10.1136/inpract.31.2.95

ROGER, P. 2009. Everyday ethics: research involving animals. *In Practice,* 31**,** 359-359. https://doi.org/10.1136/inpract.31.7.359

ROGER, P. 2011. Everyday ethics: irresponsible dog ownership. *In Practice,* 33**,** 422-422. https://doi.org/10.1136/inp.d4961

ROGER, P. 2012. Everyday ethics: promoting personal views in practice. *In Practice,* 34**,** 54-54. https://doi.org/10.1136/inp.d7470

ROGER, P. 2014. Everyday ethics: A stitch in time. In Practice, 36, 310. https://doi.org/10.1136/inp.g3485

ROGER, P. 2014. Everyday ethics: Little nippers. *In Practice,* 36**,** 430. https://doi.org/10.1136/inp.g4915

ROGER, P. 2015. Everyday ethics: Value and treatment. *In Practice,* 37**,** 47. https://doi.org/10.1136/inp.h114

ROLLIN, B. 1993. Veterinary medical ethics. *The Canadian Veterinary Journal = La revue veterinaire canadienne,* 34**,** 10-11. 17424140

ROLLIN, B. 1993. Veterinary medical ethics. *The Canadian Veterinary Journal = La revue veterinaire canadienne,* 34**,** 136-137. 17424181

ROLLIN, B. 1993. Veterinary medical ethics. *The Canadian Veterinary Journal = La revue veterinaire canadienne,* 34**,** 326-327. 17424234

ROLLIN, B. 1993. Veterinary medical ethics. *The Canadian Veterinary Journal = La revue veterinaire canadienne,* 34**,** 388-389. 17424248

ROLLIN, B. 1993. Veterinary medical ethics. *The Canadian Veterinary Journal = La revue veterinaire canadienne,* 34**,** 458-460. 17424265

ROLLIN, B. 1993. Veterinary medical ethics. *The Canadian Veterinary Journal = La revue veterinaire canadienne,* 34**,** 646-647. 17424318

ROLLIN, B. 1993. Veterinary medical ethics. *The Canadian Veterinary Journal = La revue veterinaire canadienne,* 34**,** 712-713. 17424335

ROLLIN, B. 1998. An ethicist's commentary on the dog being used to carry drugs. *The Canadian Veterinary Journal = La revue veterinaire canadienne,* 39**,** 200-201. 9559211

ROLLIN, B. 1998. Veterinary medical ethics. *The Canadian Veterinary Journal = La revue veterinaire canadienne,* 39**,** 136-137. 17424502

ROLLIN, B. 2001. Veterinary medical ethics. *The Canadian Veterinary Journal = La revue veterinaire canadienne,* 42**,** 511-513. 17424631

ROLLIN, B. 2014. Veterinary Medical Ethics Ethical Question of the Month April 2014. *The Canadian Veterinary Journal = La revue veterinaire canadienne,* 55**,** 306-307. 24688131

ROLLIN, B. E. 1991. Veterinary medical ethics. *The Canadian Veterinary Journal = La revue veterinaire canadienne,* 32**,** 4-6. 17423721

ROLLIN, B. E. 1991. Veterinary medical ethics. *The Canadian Veterinary Journal = La revue veterinaire canadienne,* 32**,** 68-69. 17423741

ROLLIN, B. E. 1991. Veterinary medical ethics. *The Canadian Veterinary Journal = La revue veterinaire canadienne,* 32**,** 138-139. 17423751

ROLLIN, B. E. 1991. Veterinary medical ethics. *The Canadian Veterinary Journal = La revue veterinaire canadienne,* 32**,** 202-203. 17423765

ROLLIN, B. E. 1991. Veterinary medical ethics. *The Canadian Veterinary Journal = La revue veterinaire canadienne,* 32**,** 327-329. 17423798

ROLLIN, B. E. 1991. Veterinary medical ethics. *The Canadian Veterinary Journal = La revue veterinaire canadienne,* 32**,** 456-457. 17423837

ROLLIN, B. E. 1991. Veterinary medical ethics. *The Canadian Veterinary Journal = La revue veterinaire canadienne,* 32**,** 522-524. 17423856

ROLLIN, B. E. 1991. Veterinary medical ethics. *The Canadian Veterinary Journal = La revue veterinaire canadienne,* 32**,** 584-585. 17423871

ROLLIN, B. E. 1991. Veterinary medical ethics. *The Canadian Veterinary Journal = La revue veterinaire canadienne,* 32**,** 648-649. 17423893

ROLLIN, B. E. 1991. Veterinary medical ethics. *The Canadian Veterinary Journal = La revue veterinaire canadienne,* 32**,** 714-715. 17423911

ROLLIN, B. E. 1992. Veterinary medical ethics. *The Canadian Veterinary Journal = La revue veterinaire canadienne,* 33**,** 7-8. 17423936

ROLLIN, B. E. 1992. Veterinary medical ethics. *The Canadian Veterinary Journal = La revue veterinaire canadienne,* 33**,** 84-85. 17423957

ROLLIN, B. E. 1992. Veterinary medical ethics. *The Canadian Veterinary Journal = La revue veterinaire canadienne,* 33**,** 296-297. 17423997

ROLLIN, B. E. 1992. Veterinary medical ethics. *The Canadian Veterinary Journal = La revue veterinaire canadienne,* 33**,** 358-359. 17424016

ROLLIN, B. E. 1992. Veterinary medical ethics. *The Canadian Veterinary Journal = La revue veterinaire canadienne,* 33**,** 422-423. 17424034

ROLLIN, B. E. 1994. An ethicists commentary on annual rabies vaccination. *The Canadian Veterinary Journal = La revue veterinaire canadienne,* 35**,** 72-73. 8069829

ROLLIN, B. E. 1994. An ethicist's commentary on the case of a breeder perpetuating a line of dogs having seizures. *The Canadian Veterinary Journal = La revue veterinaire canadienne,* 35**,** 332-333. 8069834

ROLLIN, B. E. 1994. An ethicist's commentary on the case of the dog with a persistent cough. *The Canadian Veterinary Journal = La revue veterinaire canadienne,* 35**,** 745-746. 9132285

ROLLIN, B. E. 1994. An ethicist's commentary on the case of the ewes in distress. *The Canadian Veterinary Journal = La revue veterinaire canadienne,* 35**,** 679-679. 7866958

ROLLIN, B. E. 1994. An ethicist's commentary on the case of the irresponsible veterinarian-breeder. *The Canadian Veterinary Journal = La revue veterinaire canadienne,* 35**,** 6-7. 8044765

ROLLIN, B. E. 1994. An ethicist's commentary on the case of whether a biting dog should be adopted out or destroyed. *The Canadian Veterinary Journal = La revue veterinaire canadienne,* 35**,** 598-598. 7994701

ROLLIN, B. E. 1994. An ethicist's commentary on the government swine disease policy. *The Canadian Veterinary Journal = La revue veterinaire canadienne,* 35**,** 136-137. 8055425

ROLLIN, B. E. 1995. An ethicist's commentary on the case of a client who refuses euthanasia for a sick cat. *The Canadian Veterinary Journal = La revue veterinaire canadienne,* 36**,** 471-472. 7585430

ROLLIN, B. E. 1995. An ethicist's commentary on the case of the female veterinarian receiving unwelcome attention from her employer. *The Canadian Veterinary Journal = La revue veterinaire canadienne,* 36**,** 353-353. 7648538

ROLLIN, B. E. 1995. An ethicist's commentary on the case of the recent graduate who is offended by conversations between employers. *The Canadian Veterinary Journal = La revue veterinaire canadienne,* 36**,** 410-411. 7585416

ROLLIN, B. E. 1995. An ethicist's commentary on the misdiagnosed radiograph of a racing thoroughbred. *The Canadian Veterinary Journal = La revue veterinaire canadienne,* 36**,** 739-741. 8748442

ROLLIN, B. E. 1995. An ethicist's commentary on whether a veterinarian should inform a client that a previous practitioner had left a sponge in a dog. *The Canadian Veterinary Journal = La revue veterinaire canadienne,* 36**,** 599-600. 8640632

ROLLIN, B. E. 1995. An ethicist's commentary on whether rabies vaccine for livestock should be sold over the counter. *The Canadian Veterinary Journal = La revue veterinaire canadienne,* 36**,** 178-178. 7757926

ROLLIN, B. E. 1995. An ethicist's commentary on whether veterinarians employed by large corporate entities can practise ethically. *The Canadian Veterinary Journal = La revue veterinaire canadienne,* 36**,** 74-75. 7728733

ROLLIN, B. E. 1995. An ethicist's commentary on whether veterinarians should prescribe medications that are neither therapeutic nor prophylactic to increase productivity. *The Canadian Veterinary Journal = La revue veterinaire canadienne,* 36**,** 535-536. 7497423

ROLLIN, B. E. 1995. Veterinary Medical Ethics. *The Canadian Veterinary Journal,* 36**,** 268-270. PMC1686897

ROLLIN, B. E. 1996. An ethicist's commentary on how the veterinarian should deal with an injured, unowned dog. *The Canadian Veterinary Journal = La revue veterinaire canadienne,* 37**,** 74-76. 8640654

ROLLIN, B. E. 1996. An ethicist's commentary on the case of a veterinarian who suspects an emergency clinician was negligent in treating a trauma case. *The Canadian Veterinary Journal = La revue veterinaire canadienne,* 37**,** 201-202. 8801014

ROLLIN, B. E. 1996. An ethicist's commentary on the case of the veterinarian failing to prescribe prescription drugs and losing practice income. *The Canadian Veterinary Journal = La revue veterinaire canadienne,* 37**,** 330-331. 8689592

ROLLIN, B. E. 1996. An ethicist's commentary on the case of the veterinarian who euthanized an animal thinking that he had received owner permission. *The Canadian Veterinary Journal = La revue veterinaire canadienne,* 37**,** 519-520. 8877037

ROLLIN, B. E. 1996. An ethicist's commentary on the case of the veterinarian who suspects a local cattery of being a source of feline infectious peritonitis. *The Canadian Veterinary Journal = La revue veterinaire canadienne,* 37**,** 7-8. 8746413

ROLLIN, B. E. 1996. An ethicist's commentary on the case of the veterinarian who will not utilize the proper regimen for control of roundworms. *The Canadian Veterinary Journal = La revue veterinaire canadienne,* 37**,** 585-586. 8896873

ROLLIN, B. E. 1996. An ethicist's commentary on the case of whether a veterinarian prescribing and selling drugs is in a conflict of interest situation. *The Canadian Veterinary Journal = La revue veterinaire canadienne,* 37**,** 713-714. 9111690

ROLLIN, B. E. 1996. An ethicist's commentary on whether a veterinarian should write a prescription of a branded drug in return for a financial incentive. *The Canadian Veterinary Journal = La revue veterinaire canadienne,* 37**,** 149-149. 8681280

ROLLIN, B. E. 1997. An ethicist's commentary on the case of suspected poisoning. *The Canadian Veterinary Journal = La revue veterinaire canadienne,* 38**,** 6-7. 8993779

ROLLIN, B. E. 1997. An ethicist's commentary on the case of the farmer advised by "experts". *The Canadian Veterinary Journal = La revue veterinaire canadienne,* 38**,** 745-746. 9426940

ROLLIN, B. E. 1997. An ethicist's commentary on the mismanaged cesarean section. *The Canadian Veterinary Journal = La revue veterinaire canadienne,* 38**,** 682-682. 9360787

ROLLIN, B. E. 1997. Veterinary Medical Ethics. *The Canadian Veterinary Journal,* 38**,** 407-407. PMC1576736

ROLLIN, B. E. 1998. An ethicist's commentary on the case of the elderly client seeking "unnecessary" medical advice. *The Canadian Veterinary Journal = La revue veterinaire canadienne,* 39**,** 264-265. 9592614

ROLLIN, B. E. 1998. Veterinary Medical Ethics. *The Canadian Veterinary Journal,* 39**,** 399-400. PMC1539532

ROLLIN, B. E. 1998. Veterinary Medical Ethics. *The Canadian Veterinary Journal,* 39**,** 465-466. PMC1539418

ROLLIN, B. E. 1998. Veterinary Medical Ethics. *The Canadian Veterinary Journal,* 39**,** 530-531. PMC1539424

ROLLIN, B. E. 1998. Veterinary Medical Ethics. *The Canadian Veterinary Journal,* 39**,** 676-677. PMC1539470

ROLLIN, B. E. 1999. Veterinary Medical Ethics. *The Canadian Veterinary Journal,* 40**,** 9-10. PMC1539640

ROLLIN, B. E. 1999. Veterinary Medical Ethics. *The Canadian Veterinary Journal,* 40**,** 95-96. PMC1539556

ROLLIN, B. E. 1999. Veterinary Medical Ethics. *The Canadian Veterinary Journal,* 40**,** 153-154. PMC1539672

ROLLIN, B. E. 1999. Veterinary Medical Ethics. *The Canadian Veterinary Journal,* 40**,** 222-223. PMC1539703

ROLLIN, B. E. 1999. Veterinary Medical Ethics. *The Canadian Veterinary Journal,* 40**,** 298-299. PMC1539793

ROLLIN, B. E. 1999. Veterinary Medical Ethics. *The Canadian Veterinary Journal,* 40**,** 383-384. PMC1539727

ROLLIN, B. E. 1999. Veterinary Medical Ethics. *The Canadian Veterinary Journal,* 40**,** 458-459. PMC1539745

ROLLIN, B. E. 1999. Veterinary Medical Ethics. *The Canadian Veterinary Journal,* 40**,** 535-536. PMC1539772

ROLLIN, B. E. 1999. Veterinary Medical Ethics. *The Canadian Veterinary Journal,* 40**,** 616-617. PMC1539858

ROLLIN, B. E. 1999. Veterinary Medical Ethics. *The Canadian Veterinary Journal,* 40**,** 760-761. PMC1539988

ROLLIN, B. E. 2000. An ethicist's commentary on how to decide whether a given practice is unethical. *The Canadian Veterinary Journal,* 41**,** 172-173. PMC1476324

ROLLIN, B. E. 2000. An ethicist's commentary on rabies vaccinations of raccoons. *The Canadian Veterinary Journal,* 41**,** 270-271. PMC1476160

ROLLIN, B. E. 2000. An ethicist's commentary on research protocols requiring significant animal suffering. *The Canadian Veterinary Journal,* 41**,** 748-748. PMC1476366

ROLLIN, B. E. 2000. An ethicist's commentary on the case of a proposed farm tour while cattle are infected with Salmonella. *The Canadian Veterinary Journal,* 41**,** 6-6. PMC1476338

ROLLIN, B. E. 2000. An ethicist's commentary on the ethics of painless killing. *The Canadian Veterinary Journal = La revue veterinaire canadienne,* 41**,** 662-663. 10992984

ROLLIN, B. E. 2000. Veterinary Medical Ethics. *The Canadian Veterinary Journal,* 41**,** 90-91. PMC1476276

ROLLIN, B. E. 2001. An ethicist's commentary on animal welfare versus food safety in collecting antler velvet. *The Canadian Veterinary Journal,* 42**,** 330-331. PMC1476509

ROLLIN, B. E. 2001. An ethicist's commentary on giving an analgesic to mask pain in a horse. *The Canadian Veterinary Journal = La revue veterinaire canadienne,* 42**,** 420-421. 11424574

ROLLIN, B. E. 2001. An ethicist's commentary on shooting farmed deer. *The Canadian Veterinary Journal,* 42**,** 598-598. PMC1476561

ROLLIN, B. E. 2001. An ethicist's commentary on the case of a client trying to ship a downer animal. *The Canadian Veterinary Journal,* 42**,** 760-761. PMC1476581

ROLLIN, B. E. 2001. An ethicist's commentary on the ethics of killing healthy animals. *The Canadian Veterinary Journal = La revue veterinaire canadienne,* 42**,** 908-908. 11769616

ROLLIN, B. E. 2001. An ethicist's commentary on the producer who is unwilling to euthanize sick pigs. *The Canadian Veterinary Journal,* 42**,** 8-8. PMC1476417

ROLLIN, B. E. 2001. An ethicist's commentary on the swine infected with antimicrobial-resistant Salmonella. *The Canadian Veterinary Journal,* 42**,** 88-89. PMC1476492

ROLLIN, B. E. 2001. An ethicist's commentary on whether animals raised in confinement are thus happy in confinement. *The Canadian Veterinary Journal = La revue veterinaire canadienne,* 42**,** 676-676. 11565368

ROLLIN, B. E. 2001. Veterinary medical ethics. *The Canadian Veterinary Journal = La revue veterinaire canadienne,* 42**,** 169-171. 17424617

ROLLIN, B. E. 2002. An ethicist's commentary on animal rights versus welfare. *The Canadian Veterinary Journal = La revue veterinaire canadienne,* 43**,** 913-913. 12561686

ROLLIN, B. E. 2002. An ethicist's commentary on equating productivity and welfare. *The Canadian Veterinary Journal,* 43**,** 83-83. PMC339160

ROLLIN, B. E. 2002. An ethicist's commentary on extra-label drug use. *The Canadian Veterinary Journal = La revue veterinaire canadienne,* 43**,** 749-750. 12395756

ROLLIN, B. E. 2002. An ethicist's commentary on guarding against conflict of interest. *The Canadian Veterinary Journal,* 43**,** 249-250. PMC339219

ROLLIN, B. E. 2002. An ethicist's commentary on technicians performing management procedures on farm without on-site supervision. *The Canadian Veterinary Journal = La revue veterinaire canadienne,* 43**,** 583-584. 12170833

ROLLIN, B. E. 2002. An ethicist's commentary on the case of the client requesting anaesthesia medication. *The Canadian Veterinary Journal,* 43**,** 827-827. PMC339745

ROLLIN, B. E. 2002. An ethicist's commentary on the case of the company offering a rebate if the veterinarian changes vaccines. *The Canadian Veterinary Journal,* 43**,** 411-411. PMC339285

ROLLIN, B. E. 2002. An ethicist's commentary on the mishandling of an injured animal by a raptor rehabilitation facility. *The Canadian Veterinary Journal,* 43**,** 666-667. PMC339533

ROLLIN, B. E. 2002. An ethicist's commentary on veterinarians producing autogenous vaccines and compounding antimicrobial drugs. *The Canadian Veterinary Journal,* 43**,** 164-164. PMC339184

ROLLIN, B. E. 2003. An ethicist's commentary on a client publishing your anthelminthic regimen without permission. *The Canadian Veterinary Journal,* 44**,** 276-276. PMC372232

ROLLIN, B. E. 2003. An ethicist's commentary on funding animals for continuing education. *The Canadian Veterinary Journal = La revue veterinaire canadienne,* 44**,** 867-868. 14664350

ROLLIN, B. E. 2003. An ethicist's commentary on sharing new prevention and treatment regimens. *The Canadian Veterinary Journal,* 44**,** 11-12. PMC340002

ROLLIN, B. E. 2003. An ethicist's commentary on the case of the client seeking antibiotic in feed. *The Canadian Veterinary Journal,* 44**,** 703-703. PMC340254

ROLLIN, B. E. 2003. An ethicist's commentary on veterinary "Nurse Practitioners". *The Canadian Veterinary Journal,* 44**,** 107-109. PMC340033

ROLLIN, B. E. 2003. An ethicist's commentary on when a veterinarian can render medical assistance to people. *The Canadian Veterinary Journal,* 44**,** 189-191. PMC340065

ROLLIN, B. E. 2003. An ethicist's commentary on whether veterinarians should support activist groups. *The Canadian Veterinary Journal = La revue veterinaire canadienne,* 44**,** 955-955. 14703081

ROLLIN, B. E. 2004. An ethicist's commentary on good versus natural death. *The Canadian Veterinary Journal = La revue veterinaire canadienne,* 45**,** 806-806. 15532880

ROLLIN, B. E. 2004. An ethicist's commentary on pigs who eat rodenticide. *The Canadian Veterinary Journal = La revue veterinaire canadienne,* 45**,** 643-644. 15368738

ROLLIN, B. E. 2004. An ethicist's commentary on placing animals in less-than perfect homes. *The Canadian Veterinary Journal = La revue veterinaire canadienne,* 45**,** 291-292. 15144101

ROLLIN, B. E. 2004. An ethicist's commentary on the case of a client who demands treatment by a specific veterinarian. *The Canadian Veterinary Journal = La revue veterinaire canadienne,* 45**,** 727-727. 15510682

ROLLIN, B. E. 2004. An ethicist's commentary on using wood chippers to kill chickens. *The Canadian Veterinary Journal = La revue veterinaire canadienne,* 45**,** 9-9. 14992250

ROLLIN, B. E. 2004. An ethicist's commentary on whether it is wrong to modify animals to fit our production systems. *The Canadian Veterinary Journal = La revue veterinaire canadienne,* 45**,** 899-899. 15600155

ROLLIN, B. E. 2004. Veterinary Medical Ethics Ethical Question of the Month December 2003. *The Canadian Veterinary Journal,* 45**,** 195-196. PMC3085442

ROLLIN, B. E. 2004. Veterinary Medical Ethics Ethical Question of the Month February 2004. *The Canadian Veterinary Journal,* 45**,** 379-379. PMC3085444

ROLLIN, B. E. 2004. Veterinary Medical Ethics Ethical Question of the Month March 2004. *The Canadian Veterinary Journal,* 45**,** 457-457. PMC3085445

ROLLIN, B. E. 2004. Veterinary Medical Ethics Ethical Question of the Month November 2003. *The Canadian Veterinary Journal,* 45**,** 97-97. PMC3085441

ROLLIN, B. E. 2004. Veterinary Medical Ethics Ethical Question of the Month September 2004. *The Canadian Veterinary Journal,* 45**,** 975-975. PMC2751722

ROLLIN, B. E. 2005. Ethical Question of the Month November 2004. *The Canadian Veterinary Journal,* 46**,** 111-112. PMC3085336

ROLLIN, B. E. 2005. Veterinary Medical Ethics Ethical Question of the Month April 2005. *The Canadian Veterinary Journal,* 46**,** 579-579. PMC3085385

ROLLIN, B. E. 2005. Veterinary Medical Ethics Ethical Question of the Month August 2005. *The Canadian Veterinary Journal,* 46**,** 978-978.

ROLLIN, B. E. 2005. Veterinary Medical Ethics Ethical Question of the Month December 2004. *The Canadian Veterinary Journal = La revue veterinaire canadienne,* 46**,** 203-204. 15884644

ROLLIN, B. E. 2005. Veterinary Medical Ethics Ethical Question of the Month July 2005. *The Canadian Veterinary Journal,* 46**,** 867-868. PMC3085389

ROLLIN, B. E. 2005. Veterinary Medical Ethics Ethical Question of the Month May 2005. *The Canadian Veterinary Journal,* 46**,** 683-684. PMC3085387

ROLLIN, B. E. 2005. Veterinary Medical Ethics Ethical Question of the Month June 2005. *The Canadian Veterinary Journal,* 46**,** 771-771. PMC3085388

ROLLIN, B. E. 2006. Ethical Question of the Month October 2005. *The Canadian Veterinary Journal,* 47**,** 17-17. PMC3081226

ROLLIN, B. E. 2006. Veterinary Medical Ethics Ethical Question of the Month August 2006. *The Canadian Veterinary Journal,* 47**,** 741-742. PMC1524831

ROLLIN, B. E. 2006. Veterinary Medical Ethics Ethical Question of the Month December 2006. *The Canadian Veterinary Journal,* 47**,** 1157-1158. PMC1636594

ROLLIN, B. E. 2006. Veterinary Medical Ethics Ethical Question of the Month June 2006. *The Canadian Veterinary Journal,* 47**,** 518-520. PMC3093194

ROLLIN, B. E. 2006. Veterinary Medical Ethics Ethical Question of the Month March 2006. *The Canadian Veterinary Journal,* 47**,** 200-200. PMC3086693

ROLLIN, B. E. 2006. Veterinary Medical Ethics Ethical Question of the Month May 2006. *The Canadian Veterinary Journal,* 47**,** 416-418. PMC3086774

ROLLIN, B. E. 2006. Veterinary Medical Ethics Ethical Question of the Month November 2006. *The Canadian Veterinary Journal,* 47**,** 1059-1062. PMC1624924

ROLLIN, B. E. 2006. Veterinary Medical Ethics Ethical Question of the Month October 2006. *The Canadian Veterinary Journal,* 47**,** 961-963. PMC1571134

ROLLIN, B. E. 2007. Veterinary Medical Ethics Ethical Question of the Month December 2007. *The Canadian Veterinary Journal,* 48**,** 1221-1222. PMC2081986

ROLLIN, B. E. 2007. Veterinary Medical Ethics Ethical Question of the Month February 2007. *The Canadian Veterinary Journal,* 48**,** 125-126. PMC1780227

ROLLIN, B. E. 2007. Veterinary Medical Ethics Ethical Question of the Month March 2007. *The Canadian Veterinary Journal,* 48**,** 239-241. PMC1800952

ROLLIN, B. E. 2007. Veterinary Medical Ethics Ethical Question of the Month November 2007. *The Canadian Veterinary Journal,* 48**,** 1113-1114. PMC2034418

ROLLIN, B. E. 2007. Veterinary Medical Ethics Ethical Question of the Month October 2007. *The Canadian Veterinary Journal,* 48**,** 1007-1008. PMC1978287

ROLLIN, B. E. 2008. Veterinary Medical Ethics Ethical Question of the Month April 2008. *The Canadian Veterinary Journal,* 49**,** 335-336. PMC2275335

ROLLIN, B. E. 2008. Veterinary Medical Ethics Ethical Question of the Month August 2008. *The Canadian Veterinary Journal,* 49**,** 749-752. PMC2465780

ROLLIN, B. E. 2008. Veterinary Medical Ethics Ethical Question of the Month December 2008. *The Canadian Veterinary Journal,* 49**,** 1167-1170. PMC2583411

ROLLIN, B. E. 2008. Veterinary Medical Ethics Ethical Question of the Month February 2008. *The Canadian Veterinary Journal,* 49**,** 117-120. PMC2216440

ROLLIN, B. E. 2008. Veterinary Medical Ethics Ethical Question of the Month July 2008. *The Canadian Veterinary Journal,* 49**,** 633-634. PMC2430394

ROLLIN, B. E. 2008. Veterinary Medical Ethics Ethical Question of the Month June 2008. *The Canadian Veterinary Journal,* 49**,** 535-538. PMC2387256

ROLLIN, B. E. 2008. Veterinary Medical Ethics Ethical Question of the Month October 2008. *The Canadian Veterinary Journal,* 49**,** 955-957. PMC2553509

ROLLIN, B. E. 2009. Veterinary Medical Ethics Ethical Question of the Month August 2009. *The Canadian Veterinary Journal,* 50, 801-803. PMC2711464

ROLLIN, B. E. 2009. Veterinary Medical Ethics Ethical Question of the Month December 2009. *The Canadian Veterinary Journal,* 50, 1225-1226. PMC2777285

ROLLIN, B. E. 2009. Veterinary Medical Ethics Ethical Question of the Month February 2009. *The Canadian Veterinary Journal,* 50, 131-132. PMC2629416

ROLLIN, B. E. 2009. Veterinary Medical Ethics Ethical Question of the Month January 2009. *The Canadian Veterinary Journal,* 50, 13-16. PMC2603649

ROLLIN, B. E. 2009. Veterinary Medical Ethics Ethical Question of the Month July 2009. *The Canadian Veterinary Journal,* 50, 693-696. PMC2696699

ROLLIN, B. E. 2009. Veterinary Medical Ethics Ethical Question of the Month June 2009. *The Canadian Veterinary Journal,* 50, 569-570. PMC2684044

ROLLIN, B. E. 2009. Veterinary Medical Ethics Ethical Question of the Month March 2009. *The Canadian Veterinary Journal,* 50, 229-231. PMC2643445

ROLLIN, B. E. 2009. Veterinary Medical Ethics Ethical Question of the Month November 2009. *The Canadian Veterinary Journal,* 50, 1127-1132. PMC2764512

ROLLIN, B. E. 2009. Veterinary Medical Ethics Ethical Question of the Month October 2009. *The Canadian Veterinary Journal,* 50, 1013-1016. PMC2748282

ROLLIN, B. E. 2009. Veterinary Medical Ethics Ethical Question of the Month September 2009. *The Canadian Veterinary Journal,* 50, 901-904. PMC2726016

ROLLIN, B. E. 2010. Veterinary Medical Ethics Ethical Question of the Month April 2010. *The Canadian Veterinary Journal,* 51, 351-353. PMC2839820

ROLLIN, B. E. 2010. Veterinary Medical Ethics Ethical Question of the Month December 2010. *The Canadian Veterinary Journal,* 51, 1321-1324. PMC2978983

ROLLIN, B. E. 2010. Veterinary Medical Ethics Ethical Question of the Month February 2010. *The Canadian Veterinary Journal,* 51, 131-132. PMC2808278

ROLLIN, B. E. 2010. Veterinary Medical Ethics Ethical Question of the Month January 2010. *The Canadian Veterinary Journal,* 51, 27-30. PMC2797346

ROLLIN, B. E. 2010. Veterinary Medical Ethics Ethical Question of the Month July 2010. *The Canadian Veterinary Journal,* 51, 685-688. PMC2885110

ROLLIN, B. E. 2010. Veterinary Medical Ethics Ethical Question of the Month June 2010. *The Canadian Veterinary Journal,* 51, 569-572. PMC2871350

ROLLIN, B. E. 2010. Veterinary Medical Ethics Ethical Question of the Month March 2010. *The Canadian Veterinary Journal,* 51, 237-238. PMC2822367

ROLLIN, B. E. 2010. Veterinary Medical Ethics Ethical Question of the Month May 2010. *The Canadian Veterinary Journal,* 51, 447-451. PMC2857421

ROLLIN, B. E. 2010. Veterinary Medical Ethics Ethical Question of the Month November 2010. *The Canadian Veterinary Journal,* 51, 1195-1198. PMC2957026

ROLLIN, B. E. 2010. Veterinary Medical Ethics Ethical Question of the Month October 2010. *The Canadian Veterinary Journal,* 51**,** 1067-1070. PMC2942044

ROLLIN, B. E. 2011. Veterinary Medical Ethics Ethical Issue of the Month January 2011. *The Canadian Veterinary Journal,* 52**,** 13-16. PMC3003570

ROLLIN, B. E. 2011. Veterinary Medical Ethics Ethical Question of the Month April 2011. *The Canadian Veterinary Journal,* 52**,** 353-356. PMC3058646

ROLLIN, B. E. 2011. Veterinary Medical Ethics Ethical Question of the Month August 2011. *The Canadian Veterinary Journal,* 52**,** 823-826. PMC3135025

ROLLIN, B. E. 2011. Veterinary Medical Ethics Ethical Question of the Month December 2011. *The Canadian Veterinary Journal,* 52**,** 1283-1284. PMC3215459

ROLLIN, B. E. 2011. Veterinary Medical Ethics Ethical Question of the Month February 2011. *The Canadian Veterinary Journal,* 52**,** 113-114. PMC3022446

ROLLIN, B. E. 2011. Veterinary Medical Ethics Ethical Question of the Month June 2011. *The Canadian Veterinary Journal,* 52**,** 583-586. PMC3095155

ROLLIN, B. E. 2011. Veterinary Medical Ethics Ethical Question of the Month March 2011. *The Canadian Veterinary Journal,* 52**,** 227-230. PMC3039893

ROLLIN, B. E. 2011. Veterinary Medical Ethics Ethical Question of the Month May 2011. *The Canadian Veterinary Journal,* 52**,** 463-464. PMC3077996

ROLLIN, B. E. 2011. Veterinary Medical Ethics Ethical Question of the Month November 2011. *The Canadian Veterinary Journal,* 52**,** 1169-1172. PMC3196009

ROLLIN, B. E. 2011. Veterinary Medical Ethics Ethical Question of the Month September 2011. *The Canadian Veterinary Journal,* 52**,** 927-928. PMC3157066

ROLLIN, B. E. 2012. Veterinary Medical Ethics Ethical Question of the Month April 2012. *The Canadian Veterinary Journal,* 53**,** 345-346. PMC3299502

ROLLIN, B. E. 2012. Veterinary Medical Ethics Ethical Question of the Month August 2012. *The Canadian Veterinary Journal,* 53**,** 823-826. PMC3398517

ROLLIN, B. E. 2012. Veterinary Medical Ethics Ethical Question of the Month December 2012. *The Canadian Veterinary Journal,* 53**,** 1245-1246. PMC3500113

ROLLIN, B. E. 2012. Veterinary Medical Ethics Ethical Question of the Month February 2012. *The Canadian Veterinary Journal,* 53**,** 119-120. PMC3258824

ROLLIN, B. E. 2012. Veterinary Medical Ethics Ethical Question of the Month January 2012. *The Canadian Veterinary Journal,* 53**,** 17-18. PMC3239144

ROLLIN, B. E. 2012. Veterinary Medical Ethics Ethical Question of the Month March 2012. *The Canadian Veterinary Journal,* 53**,** 223-224. PMC3280774

ROLLIN, B. E. 2012. Veterinary Medical Ethics Ethical Question of the Month November 2012. *The Canadian Veterinary Journal,* 53**,** 1153-1155. PMC3474570

ROLLIN, B. E. 2013. Veterinary Medical Ethics Ethical Question of the Month April 2013. *The Canadian Veterinary Journal,* 54**,** 321-322. PMC3595932

ROLLIN, B. E. 2013. Veterinary Medical Ethics Ethical Question of the Month December 2013. *The Canadian Veterinary Journal,* 54**,** 1111-1112. PMC3831381

ROLLIN, B. E. 2013. Veterinary Medical Ethics Ethical Question of the Month February 2013. *The Canadian Veterinary Journal,* 54**,** 115-116. PMC3552584

ROLLIN, B. E. 2013. Veterinary Medical Ethics Ethical Question of the Month January 2013. *The Canadian Veterinary Journal,* 54**,** 17-19. PMC3524808

ROLLIN, B. E. 2013. Veterinary Medical Ethics Ethical Question of the Month July 2013. *The Canadian Veterinary Journal,* 54**,** 629-631. PMC3684995

ROLLIN, B. E. 2013. Veterinary Medical Ethics Ethical Question of the Month June 2013. *The Canadian Veterinary Journal,* 54**,** 531-532. PMC3659444

ROLLIN, B. E. 2013. Veterinary Medical Ethics Ethical Question of the Month March 2013. *The Canadian Veterinary Journal,* 54**,** 213-214. PMC3573625

ROLLIN, B. E. 2013. Veterinary Medical Ethics Ethical Question of the Month May 2013. *The Canadian Veterinary Journal,* 54**,** 429-430. PMC3624909

ROLLIN, B. E. 2013. Veterinary Medical Ethics Ethical Question of the Month November 2013. *The Canadian Veterinary Journal,* 54**,** 1015-1018. PMC3801277

ROLLIN, B. E. 2013. Veterinary Medical Ethics Ethical Question of the Month October 2013. *The Canadian Veterinary Journal,* 54**,** 921-922. PMC3781422

ROLLIN, B. E. 2013. Veterinary Medical Ethics Ethical Question of the Month September 2013. *The Canadian Veterinary Journal,* 54**,** 817-820. PMC3743564

ROLLIN, B. E. 2014. Veterinary Medical Ethics - Ethical Question of the Month May 2014. *The Canadian Veterinary Journal = La revue veterinaire canadienne,* 55**,** 415-416. 24790227

ROLLIN, B. E. 2014. Veterinary Medical Ethics Ethical question of the month — December 2014. *The Canadian Veterinary Journal,* 55**,** 1127-1129. PMC4231798

ROLLIN, B. E. 2014. Veterinary Medical Ethics Ethical question of the month — October 2014. *The Canadian Veterinary Journal,* 55**,** 917-919. PMC4187372

ROLLIN, B. E. 2014. Veterinary Medical Ethics Ethical question of the month — September 2014. *The Canadian Veterinary Journal,* 55**,** 813-814. PMC4137920

ROLLIN, B. E. 2014. Veterinary Medical Ethics Ethical Question of the Month August 2014. *The Canadian Veterinary Journal,* 55**,** 719-721. PMC4095959

ROLLIN, B. E. 2014. Veterinary Medical Ethics Ethical Question of the Month February 2014. *The Canadian Veterinary Journal,* 55**,** 113-115. PMC3894866

ROLLIN, B. E. 2014. Veterinary Medical Ethics Ethical Question of the Month January 2014. *The Canadian Veterinary Journal,* 55**,** 1195-1197. PMC3866848

ROLLIN, B. E. 2014. Veterinary Medical Ethics Ethical Question of the Month July 2014. *The Canadian Veterinary Journal,* 55**,** 617-618. PMC4060904

ROLLIN, B. E. 2014. Veterinary Medical Ethics Ethical Question of the Month March 2014. *The Canadian Veterinary Journal,* 55**,** 207-210. PMC3923475

ROLLIN, B. E. 2015. Veterinary Medical Ethics Ethical question of the month — April 2015. *The Canadian Veterinary Journal,* 56**,** 327-332. PMC4357903

ROLLIN, B. E. 2015. Veterinary Medical Ethics Ethical question of the month — August 2015. *The Canadian Veterinary Journal,* 56**,** 793-795. PMC4502845

ROLLIN, B. E. 2015. Veterinary Medical Ethics Ethical question of the month — July 2015. *The Canadian Veterinary Journal,* 56**,** 651-653. PMC4466815

ROLLIN, B. E. 2015. Veterinary Medical Ethics Ethical question of the month — June 2015. *The Canadian Veterinary Journal,* 56**,** 541-543. PMC4431147

ROLLIN, B. E. 2015. Veterinary Medical Ethics Ethical question of the month — March 2015. *The Canadian Veterinary Journal,* 56**,** 223-225. PMC4327131

ROLLIN, B. E. 2015. Veterinary Medical Ethics Ethical question of the month — May 2015. *The Canadian Veterinary Journal,* 56**,** 439-442. PMC4399726

ROLLIN, B. E. 2015. Veterinary Medical Ethics Ethical question of the month — November 2015. *The Canadian Veterinary Journal,* 56**,** 1117-1118. PMC4608462

ROLLIN, B. E. 2015. Veterinary Medical Ethics Ethical question of the month — October 2015. *The Canadian Veterinary Journal,* 56**,** 1013-1014. PMC4572813

ROLLIN, B. E. 2015. Veterinary Medical Ethics Ethical question of the month — September 2015. *The Canadian Veterinary Journal,* 56**,** 909-911. PMC4535503

ROLLIN, B. E. 2016. Veterinary Medical Ethics Ethical question of the month — April 2016. *The Canadian Veterinary Journal,* 57**,** 349-350. PMC4790224

ROLLIN, B. E. 2016. Veterinary Medical Ethics Ethical question of the month — August 2016. *The Canadian Veterinary Journal,* 57**,** 813-815. PMC4944556

ROLLIN, B. E. 2016. Veterinary Medical Ethics Ethical question of the month — December 2016. *The Canadian Veterinary Journal,* 57**,** 1221-1223. PMC5109622

ROLLIN, B. E. 2016. Veterinary Medical Ethics Ethical question of the month — February 2016. *The Canadian Veterinary Journal,* 57**,** 119-121. PMC4712986

ROLLIN, B. E. 2016. Veterinary Medical Ethics Ethical question of the month — January 2016. *The Canadian Veterinary Journal,* 57**,** 11-14. PMC4677603

ROLLIN, B. E. 2016. Veterinary Medical Ethics Ethical question of the month — July 2016. *The Canadian Veterinary Journal,* 57**,** 683-685. PMC4904805

ROLLIN, B. E. 2016. Veterinary Medical Ethics Ethical question of the month — June 2016. *The Canadian Veterinary Journal,* 57**,** 577-578. PMC4866660

ROLLIN, B. E. 2016. Veterinary Medical Ethics Ethical question of the month — March 2016. *The Canadian Veterinary Journal,* 57**,** 233-234. PMC4751761

ROLLIN, B. E. 2016. Veterinary Medical Ethics Ethical question of the month — May 2016. *The Canadian Veterinary Journal,* 57**,** 461-464. PMC4827733

ROLLIN, B. E. 2016. Veterinary Medical Ethics Ethical question of the month — October 2016. *The Canadian Veterinary Journal,* 57**,** 1025-1027. PMC5026141

ROLLIN, B. E. 2016. Veterinary Medical Ethics Ethical question of the month — September 2016. *The Canadian Veterinary Journal,* 57**,** 917-919. PMC4982561

ROLLIN, B. E. 2017. Veterinary Medical Ethics - Ethical Question of the month October 2017. *The Canadian Veterinary Journal = La revue veterinaire canadienne,* 58**,** 1025-1027. 28966352

ROLLIN, B. E. 2017. Veterinary Medical Ethics - Ethical Question of the Month September 2017. *The Canadian Veterinary Journal = La revue veterinaire canadienne,* 58**,** 897-898. 28878411

ROLLIN, B. E. 2017. Veterinary Medical Ethics Ethical question of the month — April 2017. *The Canadian Veterinary Journal,* 58**,** 333-335. PMC5347322

ROLLIN, B. E. 2017. Veterinary Medical Ethics Ethical question of the month — February 2017. *The Canadian Veterinary Journal,* 58**,** 117-119. PMC5234310

ROLLIN, B. E. 2017. Veterinary Medical Ethics Ethical question of the month — January 2017. *The Canadian Veterinary Journal,* 58**,** 15-16. PMC5157732

ROLLIN, B. E. 2017. Veterinary Medical Ethics Ethical question of the month — July 2017. *The Canadian Veterinary Journal,* 58**,** 651-653. PMC5479659

ROLLIN, B. E. 2017. Veterinary Medical Ethics Ethical question of the month — June 2017. *The Canadian Veterinary Journal,* 58**,** 541-543. PMC5432140

ROLLIN, B. E. 2017. Veterinary Medical Ethics Ethical question of the month — May 2017. *The Canadian Veterinary Journal,* 58**,** 437-439. PMC5394599

ROLLIN, B. E. 2018. Veterinary Medical Ethics. *The Canadian Veterinary Journal,* 59**,** 1261-1263. 30532283

ROLLIN, B. E. 2018. Veterinary Medical Ethics. *The Canadian Veterinary Journal = La revue veterinaire canadienne,* 59**,** 1149-1151. 30410171

ROLLIN, B. E. 2018. Veterinary Medical Ethics. *The Canadian Veterinary Journal = La revue veterinaire canadienne,* 59**,** 1045-1047. 30510307

ROLLIN, B. E. 2018. Veterinary Medical Ethics. *The Canadian Veterinary Journal = La revue veterinaire canadienne,* 59**,** 935-937. 30197436

ROLLIN, B. E. 2018. Veterinary Medical Ethics. *The Canadian Veterinary Journal = La revue veterinaire canadienne,* 59**,** 829-831. 30104772

ROLLIN, B. E. 2018. Veterinary Medical Ethics - Ethical Question of the Month April 2018. *The Canadian Veterinary Journal = La revue veterinaire canadienne,* 59**,** 345-347. 29606720

ROLLIN, B. E. 2018. Veterinary Medical Ethics - Ethical Question of the Month February 2018. *The Canadian Veterinary Journal = La revue veterinaire canadienne,* 59**,** 117-119. 29386670

ROLLIN, B. E. 2018. Veterinary Medical Ethics - Ethical Question of the Month January 2018. *The Canadian Veterinary Journal = La revue veterinaire canadienne,* 59**,** 17-18. 29302098

ROLLIN, B. E. 2018. Veterinary Medical Ethics - Ethical Question of the Month July 2018. *The Canadian Veterinary Journal = La revue veterinaire canadienne,* 59**,** 699-701. 30026617

ROLLIN, B. E. 2018. Veterinary Medical Ethics - Ethical Question of the Month March 2018. *The Canadian Veterinary Journal = La revue veterinaire canadienne,* 59**,** 223-226. 29599552

ROLLIN, B. E. 2019. Veterinary Medical Ethics: Ethical Question of the month. *The Canadian Veterinary Journal = La revue veterinaire canadienne,* 60**,** 13-16. 30651645

ROLLIN, B. E. 2019. Veterinary Medical Ethics: Ethical question of the month. *The Canadian Veterinary Journal = La revue veterinaire canadienne,* 60**,** 123-125. 30705447

ROLLIN, B. E. 2019. Veterinary Medical Ethics: Ethical question of the month. *The Canadian Veterinary Journal = La revue veterinaire canadienne,* 60**,** 229-230. 30872845

ROLLIN, B. E. 2019. Veterinary Medical Ethics: Ethical question of the month. *The Canadian Veterinary Journal = La revue veterinaire canadienne,* 60**,** 1035-1036. 31597987

ROLLIN, B. E. 2019. Veterinary Medical Ethics: Ethical question of the month. *The Canadian Veterinary Journal = La revue veterinaire canadienne,* 60**,** 821-822. PMC6625170

ROLLIN, B. E. 2019. Veterinary Medical Ethics: ethical question of the month. *The Canadian Veterinary Journal = La revue veterinaire canadienne,* 60**,** 685-686. PMC6563879

ROLLIN, B. E. 2019. Veterinary Medical Ethics: ethical question of the month. *The Canadian Veterinary Journal = La revue veterinaire canadienne,* 60**,** 1147-1148. PMC6799043

ROLLIN, B. E. 2019. Veterinary Medical Ethics: ethical question of the month. *The Canadian Veterinary Journal = La revue veterinaire canadienne*, 60, 461-462. PMID31080256

ROLLIN, B.E. 2019. Veterinary Medical Ethics: ethical question of the month. *The Canadian Veterinary Journal = La revue veterinaire canadienne*, 60, 355-356. PMID30992591

ROLLIN, B.E. 2019. Veterinary Medical Ethics: ethical question of the month. *The Canadian Veterinary Journal = La revue veterinaire canadienne*, 60, 571-572. PMID: 31156255

ROLLIN, B. E. 2019. Veterinary Medical Ethics: ethical question of the month. *The Canadian Veterinary Journal = La revue veterinaire canadienne,* 60**,** 925-926. PMC6697006

ROLLIN, B. E. 2019. Veterinary Medical Ethics: ethical question of the month. *The Canadian Veterinary Journal = La revue veterinaire canadienne,* 60**,** 1261-1262. PMC6855224

ROLLIN, B. E. 2020. Veterinary Medical Ethics. *The Canadian veterinary journal = La revue veterinaire canadienne,* 61**,** 9-10. 31892749

ROLLIN, B. E. 2020. Veterinary Medical Ethics. *The Canadian veterinary journal = La revue veterinaire canadienne,* 61**,** 119-120. 32020927

ROLLIN, B. E. 2020. Veterinary Medical Ethics: ethical question of the month. *The Canadian Veterinary Journal = La revue veterinaire canadienne*, 61, 239-230. PMID: 32165745

ROLLIN, B. E. 2020. Veterinary Medical Ethics: ethical question of the month. *The Canadian Veterinary Journal = La revue veterinaire canadienne*, 61, 349-350. PMID: 32255820

ROLLIN, B. E. 2020. Veterinary Medical Ethics: ethical question of the month. *The Canadian Veterinary Journal = La revue veterinaire canadienne,* 61**,** 463-464. PMC7155872

ROLLIN, B. E. 2020. Veterinary Medical Ethics: ethical question of the month. *The Canadian Veterinary Journal = La revue veterinaire canadienne*, 61, 573-574. PMID: 32675808

ROLLIN, B. E. 2020. Veterinary Medical Ethics: ethical question of the month. *The Canadian Veterinary Journal = La revue veterinaire canadienne*, 61, 677-678. PMID: 32655151

ROLLIN, B. E. 2020. Veterinary Medical Ethics: ethical question of the month. *The Canadian Veterinary Journal = La revue veterinaire canadienne*, 61, 817-818. PMID: 32741989

ROLLIN, B. E. 2020. Veterinary Medical Ethics: ethical question of the month. *The Canadian Veterinary Journal = La revue veterinaire canadienne*, 61, 923-924. PMID: 32879517

ROLLIN, B. E. 2020. Veterinary Medical Ethics: ethical question of the month. *The Canadian Veterinary Journal = La revue veterinaire canadienne*, 61, 1035-1036. PMID: 33012817

ROLLIN, B. E. 2020. Veterinary Medical Ethics: ethical question of the month. *The Canadian Veterinary Journal = La revue veterinaire canadienne*, 61, 1137-1138. PMID: 33149349

ROLLIN, B. E. 2020. Veterinary Medical Ethics: ethical question of the month. *The Canadian Veterinary Journal = La revue veterinaire canadienne*, 61, 1243-1244. PMID: 33299239

ROLLIN, B. E. & LUNETTA, L. 2000. An ethicist's commentary on euthanizing deaf Dalmatian puppies. *The Canadian Veterinary Journal = La revue veterinaire canadienne,* 41**,** 438-439. 10857029

ROSS, K. & ROLLIN, B. 1994. An ethicist's commentary on whether veterinarians should report cruelty. *The Canadian Veterinary Journal = La revue veterinaire canadienne,* 35**,** 408-409. 8076287

ROTH, M. & ROLLIN, B. E. 1995. An ethicist's commentary on the case of shipping animals that have experienced heavy metal toxicosis. *The Canadian Veterinary Journal = La revue veterinaire canadienne,* 36**,** 9-9. 7859224

RUMNEY, J. & ROLLIN, B. E. 2000. An ethicist's commentary on whether ineffective medication should be permitted to be sold. *The Canadian Veterinary Journal,* 41**,** 354-354. PMC1476246

RUMNEY, J. & ROLLIN, B. E. 2015. Veterinary Medical Ethics Ethical question of the month — December 2015. *The Canadian Veterinary Journal,* 56**,** 1217-1220. PMC4668808

SAMSON, J. & ROLLIN, B. E. 1996. An ethicist's commentary on the case of the veterinarian asked to euthanize a healthy dog as a result of a client's last request. *The Canadian Veterinary Journal = La revue veterinaire canadienne,* 37**,** 393-393. 8809391

SANDWITH, D. & ROLLIN, B. E. 2003. An ethicist's commentary on the "raw diet". *The Canadian Veterinary Journal = La revue veterinaire canadienne,* 44**,** 450-450. 12839239

SAYERS, I. 2014. Everyday ethics: Giraffes greater good? *In Practice,* 36**,** 214. https://doi.org/10.1136/inp.g2291

SCHORI, J. 2000. A question of conscience. *Veterinary Technician,* 21**,** 591-591. WOS:000169610600009

SCHORI, J. 2000. A question of conscience: Dogfighting. *Veterinary Technician,* 21**,** 174-175. WOS:000166603300002

SCHORI, J. 2001. A question of conscience. *Veterinary Technician,* 22**,** 256, 269. WOS:000169611300017

SCHORI-DEERY, J. 2000. A question of conscience. *Veterinary Technician,* 21**,** 402-403. WOS:000169610200008

SCHORI-DEERY, J. 2000. A question of conscience. *Veterinary Technician,* 21**,** 36-37. WOS:000084822600010

SCHORI-DEERY, J. 2001. A Question of Conscience: declawing. *Veterinary Technician,* 22**,** 376. 1669295

SILVA, S. 2011. Everyday ethics: Owner access to isolation facility webcam images. *In Practice,* 33**,** 558. https://doi.org/10.1136/inp.d6600

SLIMMON, C. & ROLLIN, B. E. 1997. An ethicist's commentary on the case of the financially stressed client. *The Canadian Veterinary Journal = La revue veterinaire canadienne,* 38**,** 603-603. 9332744

SMART, N. & ROLLIN, B. E. 1992. Veterinary medical ethics. *The Canadian Veterinary Journal = La revue veterinaire canadienne,* 33**,** 696-697. 17424111

STABURSVIK, H. 2016. Everyday ethics: Changing established protocols. *In Practice,* 38**,** 94. https://doi.org/10.1136/inp.i303

STEELE, M. 2011. Everyday ethics: regulation breach on farm. *In Practice,* 33**,** 362-363. https://doi.org/10.1136/inp.d4499

STEVENSON, M. 2010. Everyday ethics: to refer or not? *In Practice,* 32**,** 122-123. https://doi.org/10.1136/inp.c994

STOGDALE, L. & ROLLIN, B. E. 2002. An ethicist's commentary on the cloning the horse case. *The Canadian Veterinary Journal,* 43**,** 6-7. PMC339065

STOGDALE, L. & ROLLIN, B. E. 2003. An ethicist's commentary on released time for voting, etc. *The Canadian Veterinary Journal,* 44**,** 537-537. PMC340189

STOGDALE, L. & ROLLIN, B. E. 2006. Veterinary Medical Ethics Ethical Question of the Month September 2006. *The Canadian Veterinary Journal,* 47**,** 845-846. PMC1555695

STOGDALE, L. & ROLLIN, B. E. 2007. Veterinary Medical Ethics Ethical Question of the Month June 2007. *The Canadian Veterinary Journal,* 48**,** 571-574. PMC1876183

STOGDALE, L. & ROLLIN, B. E. 2007. Veterinary Medical Ethics Ethical Question of the Month May 2007. *The Canadian Veterinary Journal,* 48**,** 459-462. PMC1852594

SVENSSON, S. H. 2020. Dealing with a case of suspected drug abuse in the practice. *In Practice,* 42**,** 133. https://doi.org/10.1136/inp.m466

SWAN, P. 2006. Everyday ethics Coughing Cavalier. *In Practice,* 28**,** 104-104. https://doi.org/10.1136/inpract.28.2.104

SWAN, P. 2008. Everyday ethics: ex-client with emergency. *In Practice,* 30**,** 476-477. https://doi.org/10.1136/inpract.30.8.476

SWAN, P. 2010. Everyday ethics: Tethered dog dies. *In Practice,* 32**,** 514. https://doi.org/10.1136/inp.c5959

THOMAS, L. & ROLLIN, B. E. 2014. Veterinary Medical Ethics Ethical question of the month — November 2014. *The Canadian Veterinary Journal,* 55**,** 1021-1025. PMC4204832

TIPLADY, C. M. & LAWRIE, M. 2011. Ethical dilemma - Cassie. *Australian Veterinary Journal,* 89**,** N20-21. https://doi.org/10.1111/j.1751-0813.2011.00862.x

TIPLADY, C. M. & STEPHENS, T. 2014. Euthanasia of a greyhound. *Australian Veterinary Journal,* 92**,** N28-29.

TRAN, C. V. 2016. Cyberbullying. *In:* TRAN, C. V., DESANTIS KERR, A., BILL, R. & WALSH, J. S. (eds.) *Exploring the Gray Zone.* Purdue University Press. https://doi.org/10.2307/j.ctt163t7qs.32

TRAN, C. V. 2016. Forbid or Forget? *In:* TRAN, C. V., DESANTIS KERR, A., BILL, R. & WALSH, J. S. (eds.) *Exploring the Gray Zone.* Purdue University Press. https://doi.org/10.2307/j.ctt163t7qs.22

TRAN, C. V. 2016. Hospital Volunteer. *In:* TRAN, C. V., DESANTIS KERR, A., BILL, R. & WALSH, J. S. (eds.) *Exploring the Gray Zone.* Purdue University Press. https://doi.org/10.2307/j.ctt163t7qs.14

TRAN, C. V. 2016. How Rough is Too Rough? *In:* TRAN, C. V., DESANTIS KERR, A., BILL, R. & WALSH, J. S. (eds.) *Exploring the Gray Zone.* Purdue University Press. https://doi.org/10.2307/j.ctt163t7qs.27

TRAN, C. V. 2016. Ringworm Kitten at the Babysitters. *In:* TRAN, C. V., DESANTIS KERR, A., BILL, R. & WALSH, J. S. (eds.) *Exploring the Gray Zone.* Purdue University Press. https://doi.org/10.2307/j.ctt163t7qs.25

TRAN, C. V. 2016. Sharing the Hidden Truth. *In:* TRAN, C. V., DESANTIS KERR, A., BILL, R. & WALSH, J. S. (eds.) *Exploring the Gray Zone.* Purdue University Press. https://doi.org/10.2307/j.ctt163t7qs.7

TREMBLAY, R. & ROLLIN, B. E. 1991. Veterinary medical ethics. *The Canadian Veterinary Journal = La revue veterinaire canadienne,* 32**,** 268-269. 17423781

TURNER, A. 2015. Everyday ethics: 'Off colour' alpaca. *In Practice,* 37**,** 430. https://doi.org/10.1136/inp.h4390

UEHLINGER, F. 2013. Everyday ethics: The cow that got up. *In Practice,* 35**,** 542. https://doi.org/10.1136/inp.f5551

WALSH, D. J. 2016. A Business on the Side. *In:* DESANTIS KERR, A., BILL, R., X201C, PETE, X201D, WALSH, J. S. & TRAN, C. V. (eds.) *Exploring the Gray Zone.* Purdue University Press. https://doi.org/10.2307/j.ctt163t7qs.29

WALSH, J. 2016. Employee Leaving: What to Tell the Clients? *In:* TRAN, C. V., DESANTIS KERR, A., BILL, R. & WALSH, J. S. (eds.) *Exploring the Gray Zone.* Purdue University Press. https://doi.org/10.2307/j.ctt163t7qs.11

WALSH, J. 2016. Hiring in a Close Community. *In:* TRAN, C. V., DESANTIS KERR, A., BILL, R. & WALSH, J. S. (eds.) *Exploring the Gray Zone.* Purdue University Press. https://doi.org/10.2307/j.ctt163t7qs.24

WALSH, J. 2016. Is It Discrimination? *In:* TRAN, C. V., DESANTIS KERR, A., BILL, R. & WALSH, J. S. (eds.) *Exploring the Gray Zone.* Purdue University Press. https://doi.org/10.2307/j.ctt163t7qs.20

WALSH, J. 2016. Social Media Not as private as You Think. *In:* TRAN, C. V., DESANTIS KERR, A., BILL, R. & WALSH, J. S. (eds.) *Exploring the Gray Zone.* Purdue University Press. https://doi.org/10.2307/j.ctt163t7qs.9

WALSH, J. 2016. Well-Intentioned Friend? *In:* TRAN, C. V., DESANTIS KERR, A., BILL, R. & WALSH, J. S. (eds.) *Exploring the Gray Zone.* Purdue University Press. https://doi.org/10.2307/j.ctt163t7qs.21

WALSH, J. 2016. Who Makes the Call? *In:* TRAN, C. V., DESANTIS KERR, A., BILL, R. & WALSH, J. S. (eds.) *Exploring the Gray Zone.* Purdue University Press. https://doi.org/10.2307/j.ctt163t7qs.26

WATTS, M. 2007. Everyday ethics: Injured greyhound. *In Practice,* 29**,** 118. https://doi.org/10.1136/inpract.29.2.118

WELLS, K. 2012. Everyday ethics: accommodating cultural differences of opinion. *In Practice,* 34**,** 310-311. https://doi.org/10.1136/inp.e2846

WELSH, P. 2003. VNs and the Law What Would You Do? *Veterinary Nursing Journal,* 18**,** 117-120. https://doi.org/10.1080/17415349.2003.11013225

WENSLEY, S. 2012. Everyday ethics: Genetic testing for coat colour in cats. *In Practice,* 34**,** 174. https://doi.org/10.1136/inp.e1130

WHITING, M. 2012. Everyday ethics: Homeopathic vaccine. *In Practice,* 34**,** 430. https://doi.org/10.1136/inp.e4325

WHITING, M. 2013. Everyday ethics: Vaccinations and the Animal Welfare Act. *In Practice,* 35**,** 102. https://doi.org/10.1136/inp.f172

WHITING, T. & ROLLIN, B. E. 1992. Veterinary medical ethics. *The Canadian Veterinary Journal = La revue - veterinaire canadienne,* 33**,** 486-488. 17424052

WHITING, T. & ROLLIN, B. E. 2003. An ethicist's commentary on the dairy with bad husbandry. *The Canadian Veterinary Journal,* 44**,** 787-788. PMC340286

WHITING, T. & ROLLIN, B. E. 2017. Veterinary Medical Ethics Ethical question of the month — March 2017. *The Canadian Veterinary Journal,* 58**,** 221-222. PMC5302195

WILD, S. 2017. Legal and ethical veterinary practice: a scenario evaluation. *Veterinary Nursing Journal,* 32**,** 45-49. https://doi.org/10.1080/17415349.2016.1259833

WILLIAMS, D. 2012. Everyday ethics: offering neighbourly advice. *In Practice,* 34**,** 366-366. https://doi.org/10.1136/inp.e3232

WILLIAMS, D. 2012. Everyday ethics: repeat vaccinations. *In Practice,* 34**,** 246-247. https://doi.org/10.1136/inp.e1500

WILLIAMS, D. 2013. Everyday ethics: A corneal cat-astrophe? *In Practice,* 35**,** 46. https://doi.org/10.1136/inp.e8207

WILLIAMS, D. 2018. Everyday ethics: A night at the races. *In Practice,* 40**,** 262. https://doi.org/10.1136/inp.k2825

WILLIAMS, D. 2018. Everyday ethics: Cleft palate in boxer puppies. *In Practice,* 40**,** 214. https://doi.org/10.1136/inp.k2245

WILLIAMS, D. 2018. Everyday ethics: Is this nice for mice? *In Practice,* 40**,** 422. https://doi.org/10.1136/inp.k4477

WILLIAMS, D. 2019. Blocked cats and limited finances: an ethical obstruction? *In Practice,* 41**,** 94-5. https://doi.org/10.1136/inp.l790

WILLIAMS, D. 2019. Not a dry eye in the house: treating rescue dogs with off-licence cyclosporine. *In Practice,* 41**,** 230-1. https://doi.org/10.1136/inp.l3094

WOOD, K. & ROLLIN, B. E. 2010. Veterinary Medical Ethics Ethical Question of the Month September 2010. *The Canadian Veterinary Journal,* 51**,** 935-936. PMC2920168

WOOD, S. 2011. The professional, legal and ethical issues in VN: a scenario. *The Veterinary Nurse,* 2**,** 164-168. https://doi.org/10.12968/vetn.2011.2.3.164

WOODWARD, R. 2018. Everyday ethics: An inconsequential overdose. *In Practice,* 40**,** 366. https://doi.org/10.1136/inp.k4068

WRIGHT, B. & ROLLIN, B. E. 2004. An ethicist's commentary on cultural bias in animal use. *The Canadian Veterinary Journal = La revue veterinaire canadienne,* 45**,** 555-555. 15317389

WRIGHT, R. & ROLLIN, B. E. 1997. An ethicist's commentary on the case of the veterinarian who wishes to improve rural euthanasia. *The Canadian Veterinary Journal = La revue veterinaire canadienne,* 38**,** 334-335. 9187800

WRIGHT, R. & ROLLIN, B. E. 2008. Veterinary Medical Ethics Ethical Question of the Month November 2008. *The Canadian Veterinary Journal,* 49**,** 1067-1072. PMC2572091

WU, C. 2019. Dealing with the elderly client who can no longer manage their pet's care. *In Practice,* 41**,** 397. https://doi.org/10.1136/inp.l5726

YEATES, J. 2006. Everyday ethics: Owner's dying wish. *In Practice,* 28**,** 623-624. https://doi.org/10.1136/inpract.28.10.623

YEATES, J. 2008. Everyday ethics: Obese Dog. *In Practice,* 30**,** 173-173. https://doi.org/10.1136/inpract.30.3.173

YEATES, J. 2009. Everyday ethics: prize draw with a difference. *In Practice,* 31**,** 43-43. https://doi.org/10.1136/inpract.31.1.43

YEATES, J. 2010. Everyday ethics: to treat or not? *In Practice,* 32**,** 458-459. https://doi.org/10.1136/inp.c5054

ZURRBRIGG, K. & ROLLIN, B. E. 2011. Veterinary Medical Ethics Ethical Question of the Month October 2011. *The Canadian Veterinary Journal,* 52**,** 1047-1050. PMC3174500
